# α-Methyl-α-phenylsuccinimide ameliorates neurodegeneration in a *C. elegans* model of TDP-43 proteinopathy

**DOI:** 10.1016/j.nbd.2018.06.013

**Published:** 2018-10

**Authors:** Shi Quan Wong, Matthew G. Pontifex, Marie M. Phelan, Chandra Pidathala, Brian C. Kraemer, Jeff W. Barclay, Neil G. Berry, Paul M. O'Neill, Robert D. Burgoyne, Alan Morgan

**Affiliations:** a Department of Cellular and Molecular Physiology, Institute of Translational Medicine, University of Liverpool, Liverpool, UK; b Department of Biochemistry, Institute of Integrative Biology, University of Liverpool, Liverpool, UK; c Department of Chemistry, University of Liverpool, Liverpool, UK; d Geriatrics Research Education and Clinical Center, Seattle Veterans Affairs Puget Sound Health Care System, University of Washington Department of Medicine, Seattle, WA 98108, USA

**Keywords:** MPS, α-methyl-α-phenylsuccinimide, AD, Alzheimer's disease, ALS, Amyotrophic lateral sclerosis, BSA, Bovine serum albumin, CNS, Central nervous system, DRS, Dent's Ringer solution, FTLD, Frontotemporal lobar degeneration, MOO, Multi-objective optimisation, MPO, Multiparameter optimisation, PD, Parkinson's disease, PTZ, Pentylenetetrazole, PBS, Phosphate buffered saline, PEG, Polyethylene glycol, TDP-43, Transactive response DNA binding protein of 43 kDa, WT, Wildtype, Neurodegeneration, Amyotrophic lateral sclerosis, Frontotemporal lobar degeneration, Ethosuximide, Methsuximide, Desmethylmethsuximide, *Caenorhabditis elegans*

## Abstract

The antiepileptic drug ethosuximide has recently been shown to be neuroprotective in various *Caenorhabditis elegans* and rodent neurodegeneration models. It is therefore a promising repurposing candidate for the treatment of multiple neurodegenerative diseases. However, high concentrations of the drug are required for its protective effects in animal models, which may impact on its translational potential and impede the identification of its molecular mechanism of action. Therefore, we set out to develop more potent neuroprotective lead compounds based on ethosuximide as a starting scaffold. Chemoinformatic approaches were used to identify compounds with structural similarity to ethosuximide and to prioritise these based on good predicated blood-brain barrier permeability and *C. elegans* bioaccumulation properties. Selected compounds were initially screened for anti-convulsant activity in a *C. elegans* pentylenetetrazol-induced seizure assay, as a rapid primary readout of bioactivity; and then assessed for neuroprotective properties in a *C. elegans* TDP-43 proteinopathy model based on pan-neuronal expression of human A315T mutant TDP-43. The most potent compound screened, α-methyl-α-phenylsuccinimide (MPS), ameliorated the locomotion defects and extended the shortened lifespan of TDP-43 mutant worms. MPS also directly protected against neurodegeneration by reducing the number of neuronal breaks and cell body losses in GFP-labelled GABAergic motor neurons. Importantly, optimal neuroprotection was exhibited by external application of 50 μM MPS, compared to 8 mM for ethosuximide. This greater potency of MPS was not due to bioaccumulation to higher internal levels within the worm, based on ^1^H-nuclear magnetic resonance analysis. Like ethosuximide, the activity of MPS was abolished by mutation of the evolutionarily conserved FOXO transcription factor, *daf-16*, suggesting that both compounds act via the same neuroprotective pathway(s).

In conclusion, we have revealed a novel neuroprotective activity of MPS that is >100-fold more potent than ethosuximide. This increased potency will facilitate future biochemical studies to identify the direct molecular target(s) of both compounds, as we have shown here that they share a common downstream DAF-16-dependent mechanism of action. Furthermore, MPS is the active metabolite of another approved antiepileptic drug, methsuximide. Therefore, methsuximide may have repurposing potential for treatment of TDP-43 proteinopathies and possibly other human neurodegenerative diseases.

## Highlights

• MPS is a novel neuroprotective compound that is structurally similar to ethosuximide.
• MPS is >100-fold more potent than ethosuximide.
• MPS is the active metabolite of an approved antiepileptic drug, methsuximide.
• Methsuximide may have repurposing potential for treatment of TDP-43 proteinopathies.

## 1 Background

With the predicted growth of the global ageing population, cases of age-associated neurodegenerative diseases such as Alzheimer's disease (AD), Parkinson's disease (PD) and amyotrophic lateral sclerosis (ALS) are expected to rise (Arthur et al., 2016). However, most current therapies do not decelerate or modify disease, and efforts to develop new treatments have been met with high attrition rates. Drug development efforts have largely focused on treating each neurodegenerative condition separately, however our currently limited understanding of disease-specific pathogenic mechanisms is a major impediment to this strategy. Despite their pathological and clinical heterogeneity, several underlying features are common to apparently distinct neurodegenerative diseases, such as protein aggregation, inflammation and prion-like propagation (Smith and Mallucci, 2016; Frank-Cannon et al., 2009; Brettschneider et al., 2015). Although disease-specific targets are undoubtedly of major importance for finding treatments for neurodegeneration, these common features could potentially be targeted as a complementary more general therapeutic strategy. Moreover, compounds that confer neuroprotection in multiple neurodegenerative disease models are likely to target such common features; and therefore may be useful as novel therapies and to facilitate identification of general neuroprotective mechanisms (Chen et al., 2015a).

The nematode *Caenorhabditis elegans* is an attractive choice of organism for modelling age-related neurodegenerative diseases (Chen et al., 2015a; Li and Le, 2013; Sleigh et al., 2011). Its short lifespan of around 3 weeks means that age-dependent neurodegenerative phenotypes can be assessed quickly and without the ethical constraints associated with complex model systems such as rodents. The simple nervous system of *C. elegans* coupled with its ease of genetic manipulation facilitates the development of genetic disease models of neurodegeneration and for delineating disease processes via chemical and genetic screens. Additionally, *C. elegans* has a transparent body which enables age-dependent degeneration of fluorescently-labelled neurons to be studied in vivo. In recent years, chemical screens of approved drugs in numerous *C. elegans* models of human neurodegenerative diseases have identified a chemically-diverse set of compounds with neuroprotective effects (McCormick et al., 2013; Gutierrez-Zepeda et al., 2005; Abbas and Wink, 2010; Smith and Luo, 2003; Lublin et al., 2011; Jagota and Rajadas, 2012; Keowkase et al., 2010; Arya et al., 2009; Alavez et al., 2011; Voisine et al., 2007; Kashyap et al., 2014; Tauffenberger et al., 2013; Chen et al., 2015b). However, most of these compounds have only been assessed in a single disease model and their extent of neuroprotection across multiple neurodegenerative diseases is therefore largely unknown. One notable exception is the antiepileptic drug (AED) ethosuximide, which is still used as a first line treatment for children with absence seizures (Goren and Onat, 2007). Early, pioneering work from the Kornfeld lab discovered that ethosuximide treatment increased the lifespan of wild type *C. elegans* (Evason et al., 2005; Collins et al., 2008). Subsequently, ethosuximide was shown to confer neuroprotection in three different *C. elegans* neurodegeneration models: ALS (Tauffenberger et al., 2013), frontotemporal dementia with parkinsonism-17 (Chen et al., 2015b) and adult-onset neuronal ceroid lipofuscinosis (Chen et al., 2015b). Importantly, ethosuximide's neuroprotective effects are not limited to worms, as it has also been shown to be protective in a mammalian neuroblastoma cell culture model of Huntington's disease (Chen et al., 2015b). Furthermore, ethosuximide ameliorated neuronal death and cognitive deficits in a rat in vivo amyloid beta toxin-induced AD model (Tiwari et al., 2015), suggesting the feasibility of translating its protective effects clinically and for repurposing it as a general neuroprotective agent.

Ethosuximide has been variously suggested to exert its antiepileptic action by inhibiting T-type calcium channels (Coulter et al., 1989a; Coulter et al., 1990; Coulter et al., 1989b; Gomora et al., 2001; Lacinova et al., 2000), voltage-gated sodium channels and potassium channels (Fohlmeister et al., 1984). In contrast, neuroprotection and lifespan extension by ethosuximide appears to be independent of T-type calcium channels (Chen et al., 2015b; Evason et al., 2005) and instead has been linked to altered activity of forkhead box O (FOXO) transcription factors (Chen et al., 2015b), and the phosphoinositide 3-kinase (PI3K)/protein kinase B (AKT)/Wnt/β-catenin pathway (Tiwari et al., 2015). Nevertheless, the molecular mechanism of action of ethosuximide remains unclear and no data have been reported on the direct molecular target of this widely prescribed AED.

Identification of a drug's molecular target is classically performed by affinity chromatography methods, which involves drug derivatisation for immobilisation onto an affinity matrix (Terstappen et al., 2007). Successful identification of molecular targets using this approach typically requires femtomolar to low micromolar affinities of the drug for its specific binding protein. However, the therapeutic range of ethosuximide in human epilepsy is 280–700 μM (Goren and Onat, 2007) and its neuroprotective effects in animal models require millimolar levels of the drug (Chen et al., 2015a). This low potency of ethosuximide may explain why its direct molecular target(s) have remained unknown despite being prescribed for 60 years. We reasoned that screening for structurally similar compounds with greater potency than ethosuximide could reveal novel neuroprotective compounds with translational potential that might also be used to identify shared direct molecular targets. To this end, here we report that α-methyl-α-phenylsuccinimide (MPS), confers neuroprotection and lifespan-extension in a *C. elegans* model of TDP-43 proteinopathy at a >100-fold enhanced potency compared to ethosuximide; and establish a common downstream mechanism of action for ethosuximide and MPS via the *C. elegans* DAF-16 FOXO transcription factor.

## 2 Methods

### 2.1 Maintenance and propagation of *C. elegans* strains

*C. elegans* strains were cultured at 20 °C on Nematode Growth Media (NGM; 1 mM each of CaCl_2_ and MgSO_4_, 25 mM KH_2_PO_4_, 5 μg/mL cholesterol, and in *w*/*v* 2% agar, 0.25% peptone, and 0.3% NaCl) agar culture plates seeded with the *E. coli* strain OP50. The *C. elegans* wildtype (WT) Bristol N2, *unc-49 (e407) III*, and *daf-16 (mu86) I* strains were obtained from Caenorhabditis Genetics Center (CGC, University of Minnesota, USA), whilst the human TDP-43-expressing strain CK426 [P*snb-1*::TDP-43^A315T^, P*myo-2::*dsRED] was generated by Dr. Brian Kraemer (University of Washington, USA) (Kraemer et al., 2003; Kraemer and Schellenberg, 2007; Liachko et al., 2010a), who also provided the GABAergic neuron reporter strain CZ1200 [P*unc-25*::GFP] from Prof. Yishi Jin (University of California, San Diego, USA).

### 2.2 Drug treatment

All chemicals were obtained from Sigma Chemical Co. (St. Louis, MO), except compounds 12 and 13, which were from Enamine Ltd. (Monmouth Jct., NJ); and compound 18, which was synthesized in-house. Treatment NGM plates containing water-soluble compounds succinimide and ethosuximide were prepared from concentrated stocks in agar-free NGM which were added to molten NGM agar before pouring into petri dishes. Treatment plates containing the hydrophobic compound MPS in 0.4% dimethyl sulfoxide (DMSO) vehicle, and corresponding vehicle plates, were similarly prepared from concentrated stocks with DMSO in agar-free NGM. Freshly poured plates were stored in the dark at 4 °C after drying at room temperature overnight, and transferred to room temperature and seeded up to 4 days before use. Worms age-synchronised via bleaching were chronically-exposed to treatments on plates from the L1 larval stage onwards. L1 arrest was performed by incubating bleached worms as previously described (Kashyap et al., 2014) in M9 buffer (1 mM MgSO_4_, 3 g/L KH_2_PO_4_, 5 g/L NaCl, and 6 g/L Na_2_HPO_4_) for at least 24 h (Porta-de-la-Riva et al., 2012), followed by deposition of L1 worms onto the OP50 lawn on treatment plates. Liquid-based treatment solutions for PTZ-induced seizure assays were prepared by diluting compounds at their required concentrations in 0.1% bovine serum albumin (BSA) in Dent's Ringer solution (DRS pH 7.4; 1 mM MgCl_2_, 3 mM CaCl_2_, 6 mM KCl, 10 mM HEPES, and 140 mM NaCl) with and without 7 mg/mL pentylenetetrazol (PTZ).

### 2.3 Behavioural and lifespan assays

All assays were performed at 20 °C. Seizure assays were based on a previously-described method (Williams et al., 2004), whereby treatment with the pro-convulsant pentylenetetrazole (PTZ) induces characteristic repetitive ‘head bobbing’ seizures in worms containing null mutations in the GABA_A_-receptor-encoding gene *unc-49*. Briefly, unsynchronised 1–3-day-old adult worms were pre-incubated in liquid droplets containing ethosuximide-based compounds in DRS for 2 h prior to treatment with 7 mg/mL PTZ in DRS for 15 mins, at which point the number of ‘head bobs’ was quantified for 30 s. Seizures were defined as head bobbing movements manifested by head extension from the body followed by shrinkage back into the body, and were scored in a qualitative and quantitative manner. Qualitative scoring simply assessed whether individual worms were undergoing seizures, as defined by exhibiting at least three repetitive head bobs over the scoring duration; whereas the quantitative approach scored the total number of head bobs over the same duration. DMSO-soluble compounds were assayed at a DMSO concentration of 1%, as pretreatment with 1% DMSO did not cause any observable abnormality in the worms' behaviour, with 100% of worms seizing in response to PTZ. Toxicity was determined by visual observations of abnormal stiff movement or immobility, and a weak response to touch or death as indicated by the absence of response to touch at the end of drug pre-treatment and/or PTZ exposure. Conversely, a therapeutic effect was defined by a reduction or abolishment of seizures in the presence of movement and response to touch. If absence of seizures was observed with immobility, a non-seizing phenotype was scored only if a robust response to touch was observed, indicative of non-toxicity.

Motility and lifespan assays were performed on age-synchronised strains. Motility was assessed by quantifying the number of body bends in the unparalysed posterior half of these strains on unseeded treatment NGM plates for 1 min after 20 s of acclimatisation. Scoring was performed when sinusoidal, lateral, or coiling movements deviate maximally from and back to the original position. Lifespan assays were initiated with 60 day-1 adults which were scored for survival every alternate day, and concluded when all worms were dead. To prevent progeny contamination, worms were concurrently transferred to freshly seeded treatment plates with each assay day. Worms were scored as alive in the presence of spontaneous locomotion, locomotive responses to touch stimulation with a worm pick, and/or pharyngeal pumping. Missing and dead worms which displayed internally-hatched progenies or visible signs of physical damage such as gut spillage or gonad extrusion were censored from analysis.

### 2.4 In vivo GABAergic neuronal imaging

Live, age-synchronised worms were immobilised in polyethylene glycol (PEG)/glycerol solution in 1× phosphate buffered saline (PBS) (20% each of PEG (*w*/*v*) and glycerol) on a 22 × 40 mm imaging slide (VWR International, Pennsylvania, USA). Slides were then mounted with a 13-mm coverslip (Paul Marienfeld GmbH & Co. KG, Germany) and imaged under fluorescence with a Nikon Eclipse Ti S microscope (Nikon Instruments Europe BV, Netherlands) to visualise GFP-labelled GABAergic neurons. Quantification of neuronal cell bodies and breaks of the D-type GABAergic inhibitory motor neurons in the ventral nerve cord was carried out using NIS-Elements imaging software version 3.2 (Nikon Instruments Europe BV, Netherlands). Cell bodies were scored when intact and clear punctate fluorescence was observed, whilst neuronal breaks were quantified from breaks in the fluorescence within the neuronal subset.

### 2.5 Genetic crosses

N2 males were mated with CK426 [P*snb-1*::TDP-43^A315T^, P*myo-2::*dsRED] hermaphrodites, and resulting transgenic male offspring were crossed with CZ1200 [P*unc-25*::GFP] hermaphrodites to obtain uncoordinated hermaphrodites which express both pharyngeal dsRED and GABAergic neuronal GFP markers. Progenies from these worms were genotyped to verify presence of the human TDP-43-encoding *TARDBP* gene via PCR using primers which target the transgene [forward: 5′-CTGAATATATTCGGGTAACCG-3′; reverse: 5′- CAGCCAGAAGACTTAGAATCC-3′] and TDP-43 protein expression via immunoblotting using a mouse monoclonal anti-TDP-43 primary antibody (Abcam, Cambridge, UK) at 1:1000 dilution. This new strain was given the designation AMG619 [P*snb-1*::TDP-43^A315T^, P*myo-2::*dsRED; P*unc-25*::GFP]. To introduce a deletion in the *daf-16* allele in AMG619 worms, *daf-16 (mu86) I* hermaphrodites were first mated with N2 males and *daf-16 (mu86) I* male progenies were then crossed with AMG619 hermaphrodites. As there is no visible selection marker for the *daf-16 (mu86) I* allele, hermaphrodite progenies were selected using the fluorescent markers and uncoordinated phenotype for AMG619. Homozygosity for the *daf-16 (mu86) I* deletion was verified with PCR using primers which distinguished between the mutation [forward: 5′-CCCACATTCGTGTGGGTTTTCTAGTCG-3′; reverse: 5′-CGTTATCAAATGCTCCTTGCATTGAATC-3′] and the wildtype *daf-16* allele [forward: 5′-CCCACATTCGTGTGGGTTTTCTAGTCG-3′; reverse: 5′-GCGTCAGTTCCGATCTGATATGAAC-3′], and presence of the *TARDBP* gene was verified as before. This new strain was given the designation AMG620 [*daf-16 (mu86) I;* P*snb-1*::TDP-43^A315T^, P*myo-2::*dsRED; P*unc-25*::GFP].

### 2.6 Selection of primary screening compounds

**Fig. 1:**
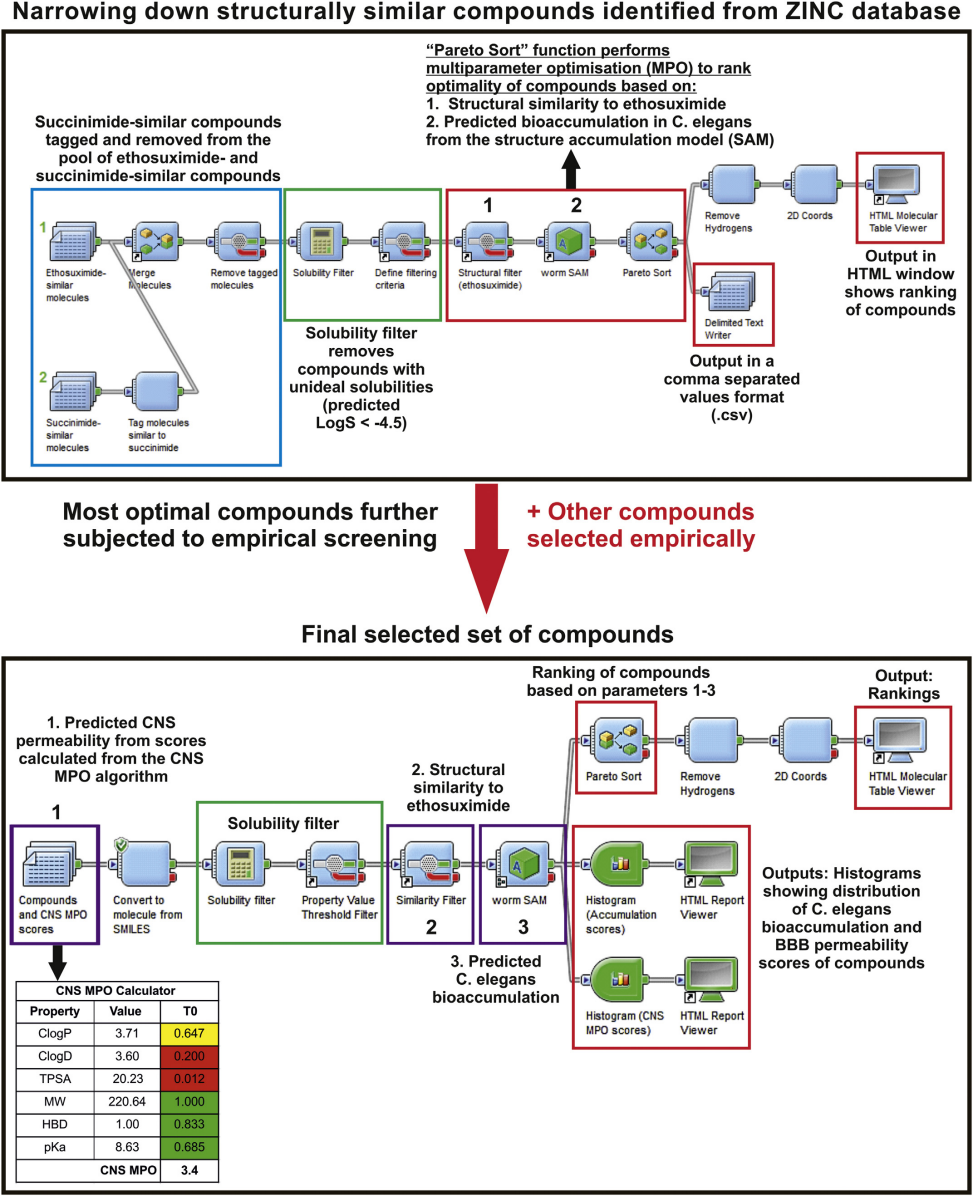
Workflow for identification of structurally-similar compounds to ethosuximide. Compounds for screening were identified and selected empirically and from the ZINC database, and were ranked for their optimality based on predicted CNS permeability (Holliday et al., 2002) and *C. elegans* bioaccumulation (Wager et al., 2010), and the extent of similarity to ethosuximide using Pipeline Pilot™.

Structurally-similar compounds to ethosuximide were selected empirically or extracted from the ZINC database version 12 (Irwin and Shoichet, 2005) based on substructure search (Barnard, 1993) and 70 to 90% molecular similarity as measured with the Tanimoto coefficient (Tc) (Holliday et al., 2002). Further compound refinement to select the most optimal molecules for screening was guided with the Accelrys Pipeline Pilot™ software version 9.0 (Accelrys Software Inc., California, USA) to rank compounds with an aqueous solubility (LogS) cut off value of −4.5 via the “Pareto Sort” function based on overall optimality for the following parameters: 1) central nervous system (CNS) permeability as predicted from the CNS multiparameter optimisation (MPO) algorithm (Wager et al., 2010), 2) Tc similarity to ethosuximide, and 3) *C. elegans* bioaccumulation propensity predicted from a worm structure-based accumulation (SAM) model (Burns et al., 2010) (Fig. 1).

### 2.7 Chemical synthesis

Compound 18 (2,5-Pyrrolidinedione,3-ethyl-1,3-dimethyl) is an *N*-methylated form of ethosuximide and was generated from ethosuximide in a single step reaction with the methylating agent iodomethane in anhydrous conditions, as adapted from a previously reported procedure (Khan et al., 2001). The reaction was initially left to react at room temperature for 3 days and continued for an additional night with half the original amount of iodomethane (6.8 mM). Analytical thin layer chromatography performed on a silica gel-coated aluminium plate was used to verify successful generation of the *N*-methylated product. The reaction mixture was then subjected to filtration and reduced pressure to remove K_2_CO_3_ and acetone, respectively. The product was purified by silica gel column chromatography at hexane/ethyl acetate = 4/1 and confirmed for its identity after solvent removal from combined appropriate fractions.

### 2.8 Measuring internal drug concentration in *C. elegans* with 1D ^1^H NMR spectroscopy

Synchronised CK426 L1 worms were cultured at 3000 worms per treatment plate seeded with 100 μL OP50. At 70 h post L1-plating, day 1 adults were washed off plates and cleaned with M9 buffer thrice through pelleting by gravity for 6 mins. Worms were transferred to Corning® Costar® Spin-X® tube filters and spun at 450 g for 1.5 mins to remove excess liquid, followed by resuspension in water and transferral to Eppendorf® LoBind microcentrifuge tubes. Five percent of the worm suspension was set aside for estimating the analysed sample size and the final worm wash solution was checked by NMR to ensure that all treatment peaks observed in the extracts originated from within the worms.

Metabolites were extracted from samples with 100 mM of the NMR reference compound sodium trimethylsilyl-^2^H_4_-propionate (TSP) in 50% (v/v) water and ice-cold acetonitrile (AcN). This was performed on an ice bath via sonication with an MSE Soniprep 150 Plus sonicator/cell disruptor (Wolf Laboratories Ltd., York, UK) at a fixed frequency of 50 kHz for three 30-s durations with 30-s rest intervals between and 10% amplitude as adapted from previously described protocols (Khan et al., 2001). Samples were vortexed for 30 s and centrifuged at 21,000 ×*g* for 5 mins at 4 °C, followed by snap-freezing of metabolite-containing supernatants in liquid nitrogen and lyophilisation to completion with a Heto PowerDry LL1500 freeze dryer (ThermoFisher Scientific Inc). Immediately prior to 1D ^1^H NMR spectroscopy analysis, 200 μL of deuterated phosphate buffer (0.0012 and 100 mM of sodium azide and sodium phosphate pH 7.4 in deuterated water (Goss Scientific Instruments, Cheshire, UK)) were added to lyophilised extracts, vortexed for 1 min, and centrifuged at 12,000 ×*g* for 2 mins at 20-25 °C. The clarified extracts were each transferred to clean 3-mm outer diameter NMR SampleJet tubes (Bruker BioSpin Gmbh) and analysed by 1D ^1^H NMR spectroscopy using a Bruker Avance III NMR spectrometer equipped with a 5 mm TCI CryoProbe with a proton resonance frequency of 600 MHz (Bruker BioSpin Gmbh). 1D ^1^H spectra were acquired at 25 °C on TopSpin™ version 3.5pl6 (Bruker BioSpin Gmbh), with the manufacturer-provided nuclear Overhauser enhancement spectroscopy (noegpr1d) and Carr-Purcell-Meiboom-Gill (cpmgpr1d) pulse sequences and automatically processed, referenced and phased using vendor supplied routines (apk0.noe). Calculation of internal compound concentrations was based on the published volume of 4 nL for a day 1 adult worm of the wild type N2 strain at approximately 85 h post-hatch (Hirose et al., 2003), based on the sum of around 15 h for development to L1-arrest after hatching at 20 °C and sample processing at 70 h post L1-arrest.

### 2.9 Statistical analysis

All general statistical analyses were performed on GraphPad Prism version 6 (GraphPad Software Inc., California, USA), except analyses and comparison of lifespan which were carried out with the log-rank test on the Online Application for the Survival Analysis of Lifespan Assays 2 (OASIS 2;(Han et al., 2016)). Concentration-response curves were fitted with GraphPad Prism 6 via nonlinear regression with the variable slope model, followed by subsequent derivation and statistical comparison of LogEC_50_ values. Data is presented as mean values, with standard error of mean when appropriate.

## 3 Results

### 3.1 Chemoinformatic selection and synthesis of ethosuximide-based compounds

In order to identify molecules with similarity to ethosuximide and prioritise these for screening in *C. elegans*, we employed computational chemistry approaches (Fig. 1). Initially, compounds similar to both ethosuximide and its biologically inert parent compound succinimide were retrieved from the ZINC database. This was performed based on both substructure search and similarity searching using Tc thresholds of at least 70%, which are generally considered to represent high similarity (Sheridan and Kearsley, 2002). Identified compounds were subjected to subsequent analysis on the Pipeline Pilot™ program to narrow down the pool so as to facilitate the selection of compounds for screening (Fig. 1). Since succinimide and ethosuximide are themselves structurally similar, and as the goal was to derive bioactive compounds similar to ethosuximide for initial screening, identified compounds similar to inert succinimide were tagged and removed such that only compounds similar to ethosuximide remained for selection. Aqueous solubility is an important parameter for drugs acting on the central nervous system (CNS), as >85 and 90% of approved CNS drugs have been found to have LogS values greater than −4 and −5mol/L, respectively (Alelyunas et al., 2010). Hence, identified ethosuximide-similar compounds that remained in the Pipeline Pilot™ analysis were therefore subjected to a solubility filter with an intermediate LogS cut-off of at least −4.5 to remove those which did not fufill this solubility criterion. Retained compounds with desirable solubilities were subsequently ranked for their overall optimality, based on structural similarity to ethosuximide and predicted *C. elegans* bioaccumulation propensity (Burns et al., 2010), by maximising for all parameters with a multiparameter optimisation (MPO) approach. Compound selection was additionally aided by empirical medicinal chemistry expertise to prioritise computationally identified and ranked compounds, and to identify existing potential chemical spaces and approved drugs with some similarity to ethosuximide which may have been excluded from initial computational detection.

**Fig. 2:**
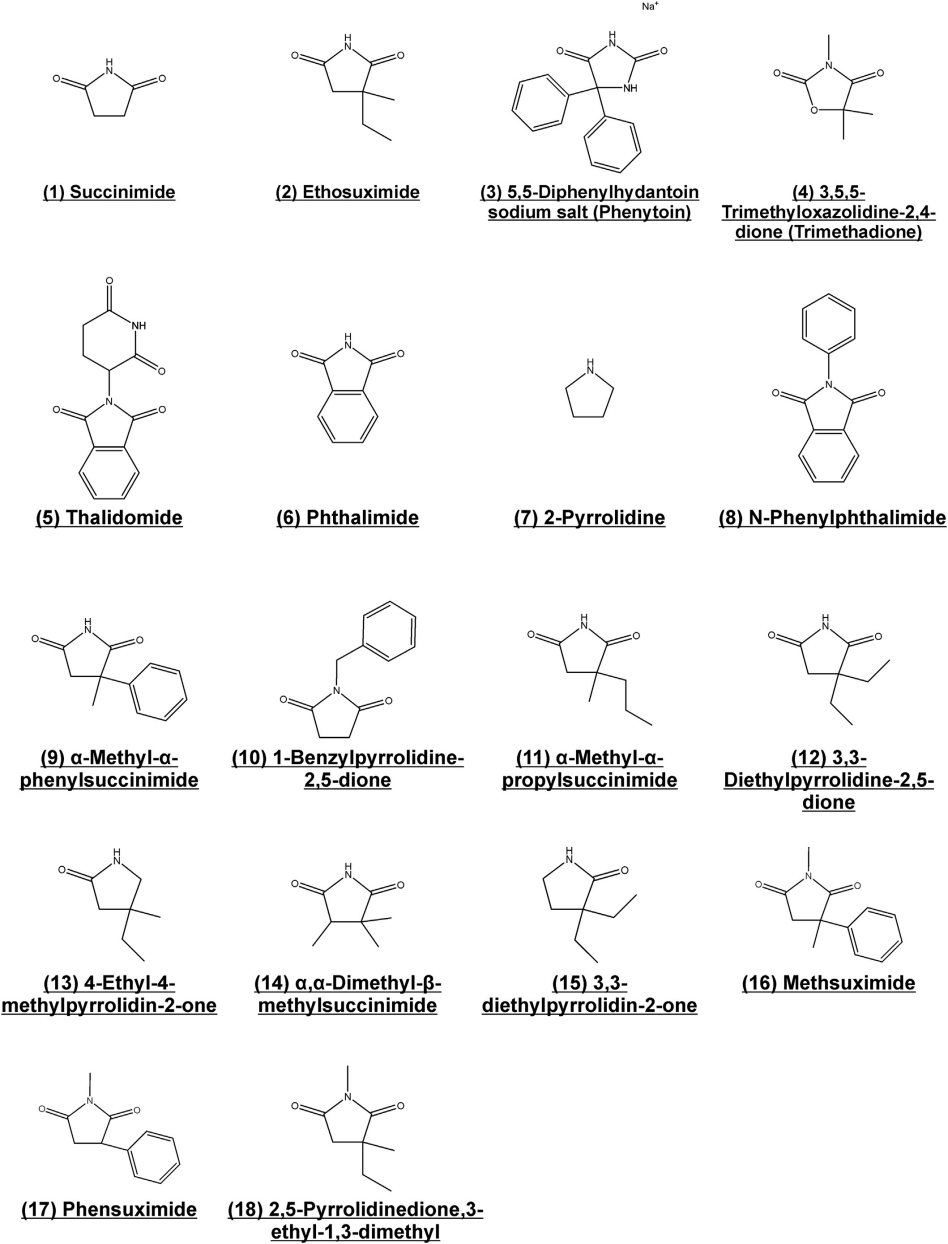
Set of compounds selected for screening. Chemoinformatic and empirical approaches were used to identify molecules with similarity to ethosuximide that had desirable properties and high predicted blood-brain barrier permeability.

A total of 18 compounds were thereby selected for screening, including ethosuximide and succinimide, which were to be utilised as positive and negative controls respectively (Fig. 2). Due to the small number of compounds analysed at this stage, the predicted CNS permeability was additionally manually curated for each compound using the CNS MPO algorithm (Wager et al., 2010), and fed into the Pipeline Pilot™ analysis as an additional MPO parameter for compound ranking. All selected compounds were predicted to possess good CNS permeability as evident from high CNS MPO scores ranging from 4.3 to a perfect score of 6 (Table S1; scores of ≥4 are considered to be high (Wager et al., 2010)). Overall the selected compounds displayed good predicted permeation of the CNS, small differences in predicted *C. elegans* bioaccumulation, and a good overall ranking, confirming their optimality for screening (Table S2). With the exception of compound 18 (2,5-Pyrrolidinedione,3-ethyl-1,3-dimethyl), all molecules were available and obtained from commercial suppliers. Compound 18 was synthesized via a single step *N*-methylation reaction of ethosuximide using iodomethane and subsequently purified by thin layer chromatography.

### 3.2 Primary screen for in vivo bioactivity in *C. elegans*

**Fig. 3:**
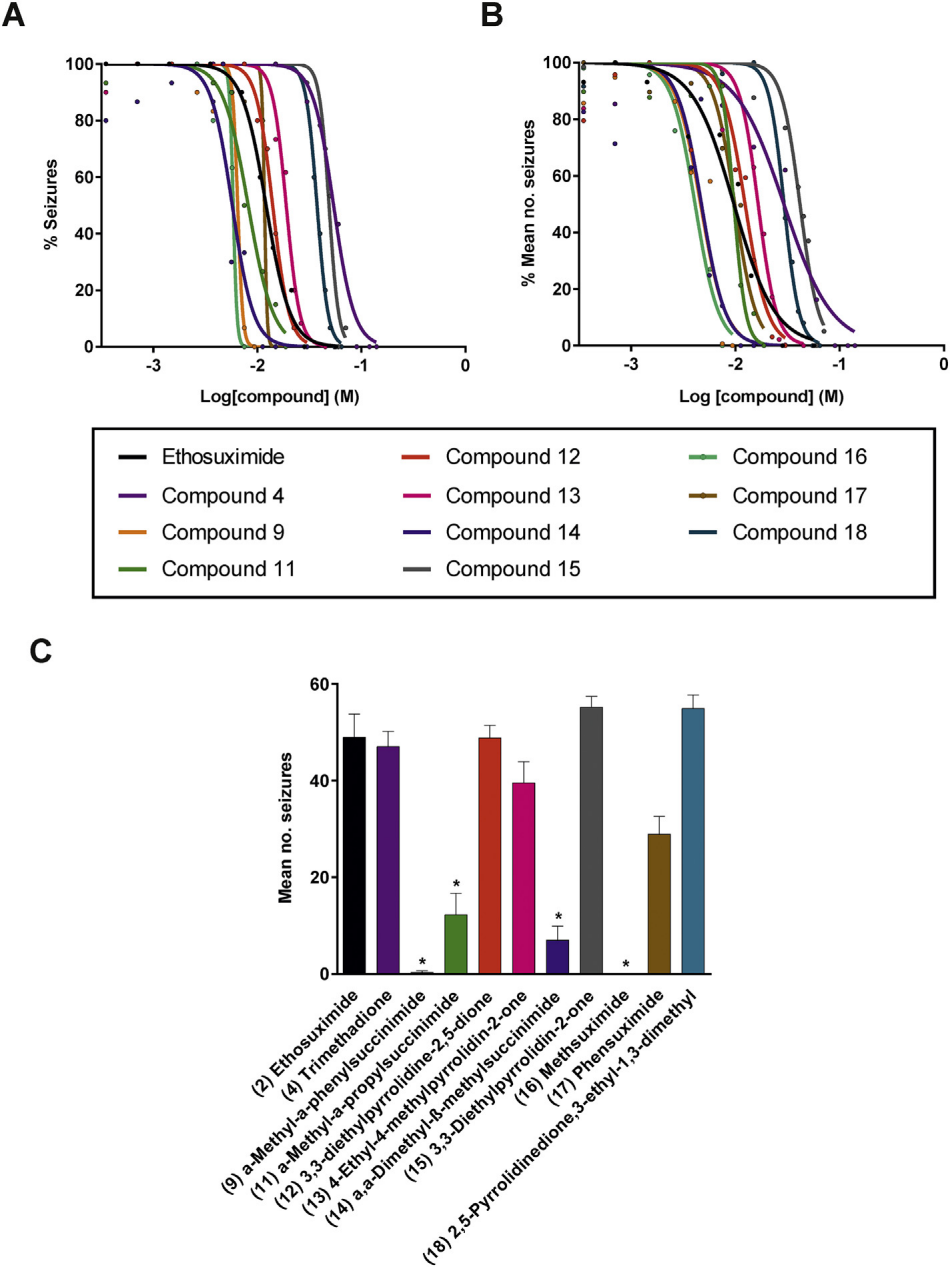
Compound screen for bioactivity using a worm seizure assay. Worms were pre-treated for 2 h with the indicated concentration of compounds and then incubated for a further 15 mins in the same solution with 7 mg/mL pentylenetetrazole added. At this point, head-bobbing seizures were assessed over a time period of 30 s. Concentration-response curves were derived from scoring of (A) percentages of seizing worms and (B) frequencies of seizures in *unc-49* worms. Significant leftward shifts of curves from compounds 9, 14, and 16 in comparison to that of ethosuximide are indicative of enhanced potencies in comparison to the drug (p < .05). Data shown was pooled from three biological replicates. Curve fit and significance of shifts, as derived from comparisons of fitted LogEC_50_ values, were performed via nonlinear regression analysis (n = 5–15 worms per concentration per compound). (C) Seizure-protecting activities of compounds was compared at a single concentration of 7.5 mM. Compounds 9, 11, 14, and 16 greatly reduced seizure rates as compared to ethosuximide at the same concentration (p < .05). Data shown was pooled from three biological replicates, with comparisons made via the Kruskal-Wallis test with Dunn's multiple comparison (n = 14–15 worms per concentration per compound); *, p < .05.

Neurodegeneration phenotypes are most evident in aged animals and involve time consuming, labour-intensive assays. As ethosuximide is an anti-epileptic drug, we took advantage of a previously described worm PTZ-induced seizure assay (Williams et al., 2004) to develop a more rapid primary screening platform to identify bioactive molecules amongst our selected set of ethosuximide-related compounds. The characteristic head-bobbing convulsions induced by PTZ in worms containing null mutations in the GABA_A_-receptor-encoding gene *unc-49* were unaffected by the biologically inert succinimide molecule (Movie S1), but were greatly reduced by the structurally-related compound ethosuximide (Movie S2). This therefore confirmed the validity of the PTZ assay for screening for bioactive anticonvulsant molecules. Several compounds were unable to be assessed due to solubility problems based on the conditions required for the assay, namely compounds 3 (phenytoin), 5 (thalidomide), 6 (phthalimide), 8 (*N*-phenylphthalimide), and 10 (1-benzylpyrrolidine-2,5-dione). The remaining compounds were first subjected to a preliminary screen against ethosuximide and succinimide at a single optimal reference ethosuximide concentration of 4 mg/mL, or 2 mg/mL in the event of observed toxicity at 4 mg/mL. This initial pre-screen identified compounds 9 (α-methyl-α-phenylsuccinimide), 11 (α-methyl-α-propylsuccinimide), 12 (3,3-diethylpyrrolidine-2,5-dione), 13 (4-ethyl-4-methylpyrrolidin-2-one), 14 (α,α-dimethyl-β-methylsuccinimide), 15 (3,3-diethylpyrrolidin-2-one), 16 (methsuximide), 17 (phensuximide), and 18 (2,5-pyrrolidinedione,3-ethyl-1,3-dimethyl) as active in reducing both the percentage of animals undergoing seizures (Fig. S1A) and the frequency of seizures (Fig. S1B). Like ethosuximide, these active compounds protected against seizures when screened at 2 or 4 mg/mL, or when re-screened at a higher concentration of 10 mg/mL if activity was not seen at the lower concentrations. Compound 7 (2-pyrrolidine) was not protective against induced seizures even at 10 mg/mL, and was therefore classified as inactive. Although the effect of compound 4 (trimethadione) on the frequency of seizures was not statistically significant at 4 mg/mL, it produced a partial reduction in the percentage of animals undergoing seizures, and so it was further assayed alongside all the active compounds across a wide range of concentrations for subsequent potency assessment (Fig. 3). The resultant concentration-response curves generally exhibited the same shift patterns from ethosuximide for both qualitative (percentage of worms seizing in the population) and quantitative (mean frequency of seizures) scoring approaches (Fig. 3A,B). Compounds 4, 11, 12, 13, 15, 17 and 18 exhibited either no significant difference from ethosuximide or were significantly less potent, as indicated by up to 4-fold higher EC_50_ values (Table S3). In contrast, compounds 9, 14 and 16 were significantly more potent, with approximately 2-fold lower EC_50_ values in comparison to ethosuximide's EC_50_ of 9.7 mM. To better emphasize these potency differences, anticonvulsive effects were compared at a single concentration of 7.5 mM (Fig. 3C). This clearly demonstrated that seizure protection by compounds 9, 14, and 16 was greatly improved in comparison to ethosuximide at the same concentration.

### 3.3 Compound 9 (α-methyl-α-phenylsuccinimide) is neuroprotective

**Fig. 4:**
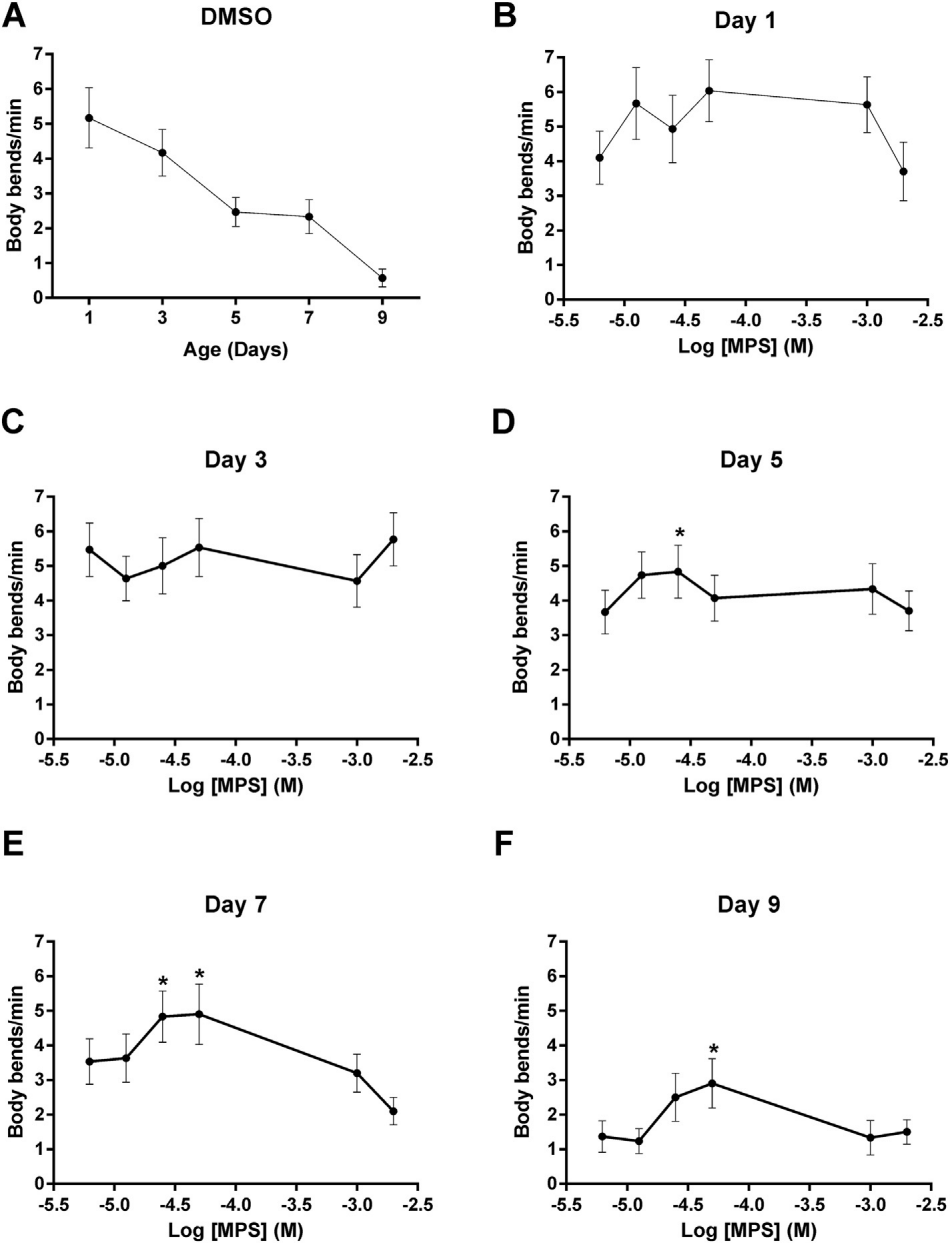
MPS concentration-response relationship using a worm TDP-43 proteinopathy model. Body bend frequencies of chronically-treated, age-synchronised TDP-43(A315T)-expressing worms were assessed to determine the concentration-response relationship of the compound at various ages of adulthood. (A) Body bends of control worms treated with the 0.4% DMSO vehicle at all ages. Although control values were not incorporated into concentration-response curves on the logarithmic scale as a logarithmic value cannot be obtained for 0 M of the compound, body bend frequencies for each day were compared against respective control values for statistical analysis. Locomotion defects were not ameliorated at any concentration on (B) days 1 and (C) 3 (p > .05). (D - F) At older ages, locomotion rate was significantly improved at concentrations of −4.6 M (0.025 mM) and −4.3 M (0.05 mM) (p < .05), although higher concentrations reduced it instead. Data shown was pooled from three biological replicates, and comparisons were made with the Kruskal-Wallis test with Dunn's multiple comparisons (n = 30 worms per day per concentration); *, p < .05.

**Fig. 5:**
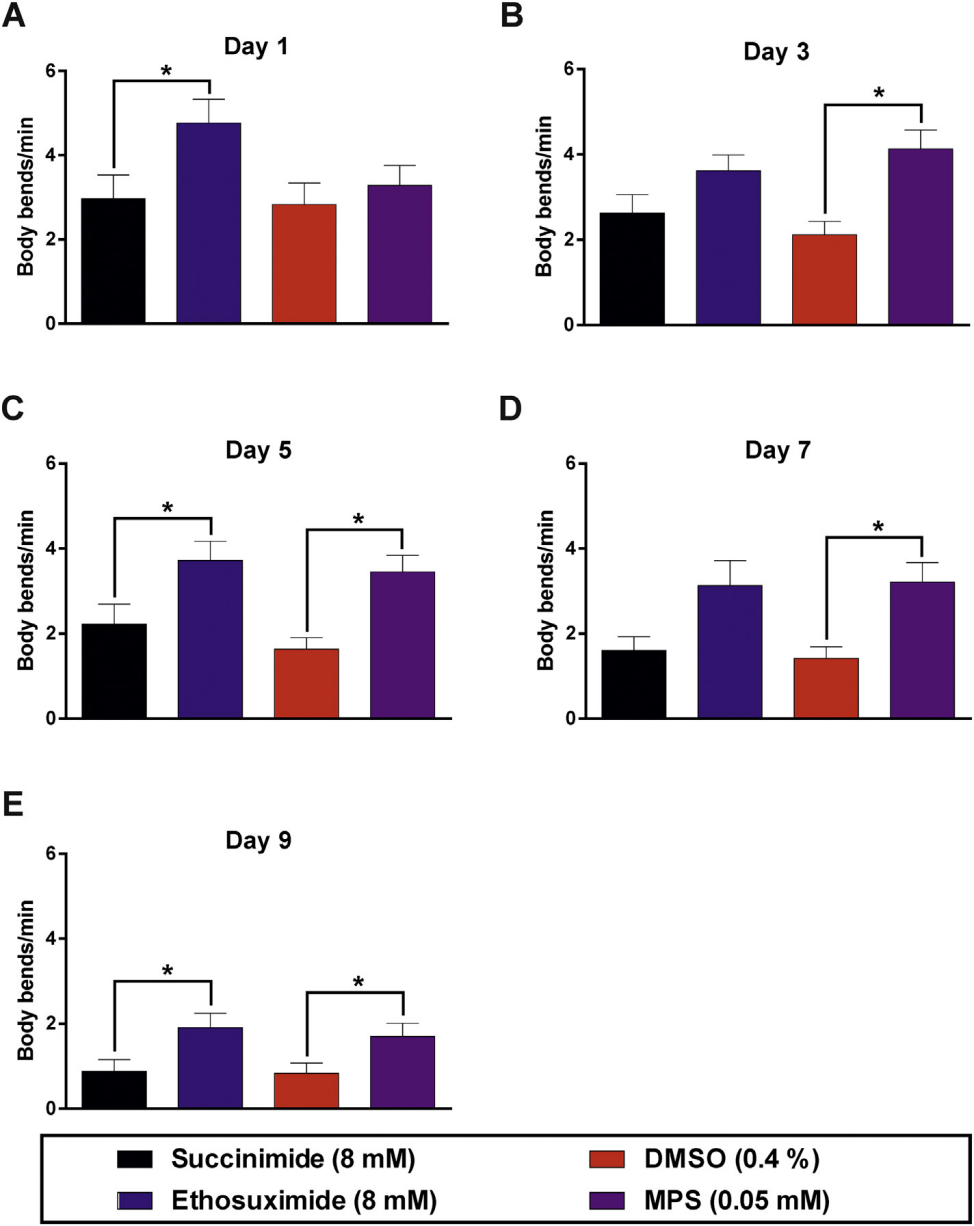
MPS ameliorates locomotion defects in the worm TDP-43 proteinopathy model. Age-synchronised worms were chronically treated with 8 mM succinimide or ethosuximide, 0.4% DMSO vehicle, or 0.05 mM MPS in 0.4% DMSO from the L1 larval stage. Ethosuximide increased the frequency of body bends per minute at most ages except days (B) 3 and (D) 7, whilst MPS consistently ameliorated locomotion defects from day 3 onwards (B-E; p < .05). Data shown was pooled from six biological replicates. Comparisons were performed via the Kruskal-Wallis test with Dunn's multiple comparisons (n = 40–60 worms per day per treatment); *, p < .05.

Of the three compounds with similarly enhanced potencies, compound 9 (α-methyl-α-phenylsuccinimide; MPS) was selected for further study, based on considerations of cost and the presence of a phenyl moiety that would facilitate future medicinal chemistry approaches to improve its potency and physicochemical properties further. Neuroprotective activity was assessed in a *C. elegans* transgenic model of TDP-43 proteinopathy (Liachko et al., 2010a). This strain pan-neuronally expresses a missense mutation in the human *TARDBP* gene that results in an A315T mutated form of the RNA-binding protein TDP-43, which is known to cause familial ALS (Gitcho et al., 2008; Sreedharan et al., 2008). Importantly, the impaired movement, reduced lifespan and neurodegeneration exhibited by worms expressing pan-neuronal TDP-43(A315T) has previously been shown to be ameliorated by ethosuximide (Tauffenberger et al., 2013), thereby justifying its use. We first determined the effect of a range of concentrations of MPS on locomotion, by quantifying the frequency of body bends on agar. MPS increased locomotion in worms of over 3 days of age, with the most consistent significant effect observed at 0.05 mM (Fig. 4). We therefore compared the effect of 0.05 mM MPS to that of ethosuximide at its previously determined optimal neuroprotective concentration of 8 mM (Chen et al., 2015b). At these concentrations, ethosuximide significantly increased the frequency of body bends in TDP-43(A315T)-expressing worms from day 1 of age, albeit this protective effect was not consistently observed at all ages assessed; whereas significant improvements of locomotion by MPS was consistently exhibited from 3 days of age (Fig. 5).

**Fig. 6:**
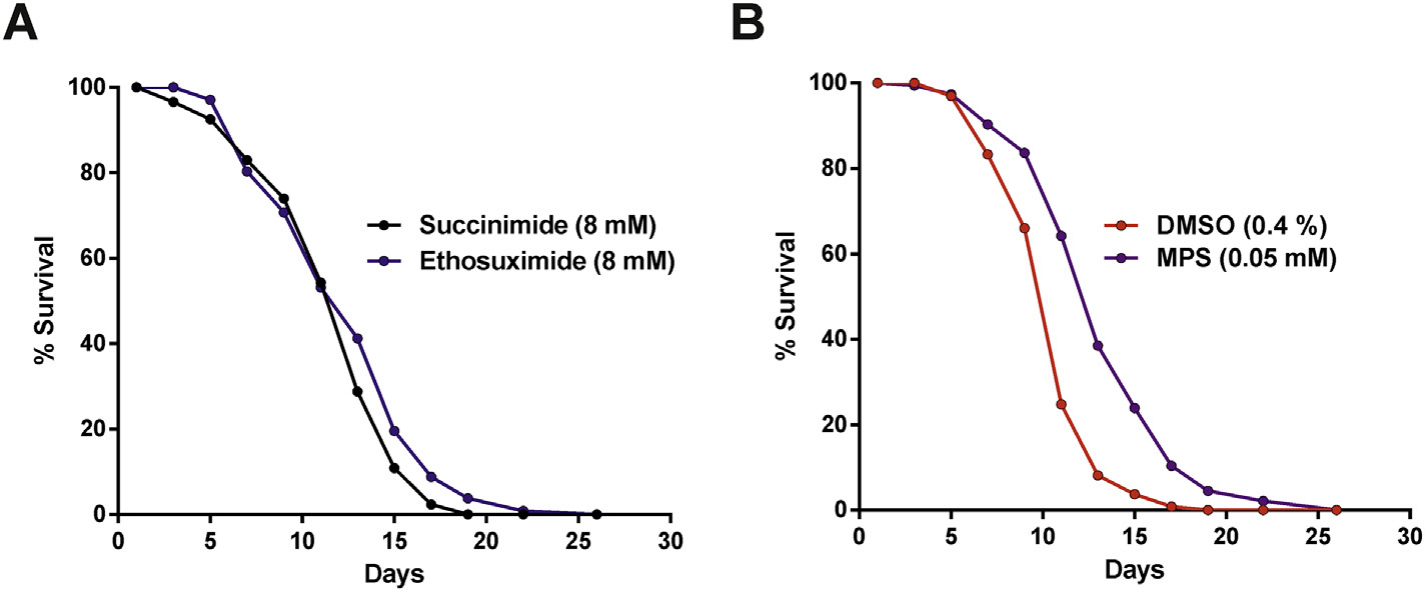
MPS extends lifespan in the worm TDP-43 proteinopathy model. Lifespan analyses were performed on age-synchronised worms chronically treated from the L1 larval stage onwards. Worms were scored as dead in the absence of pharyngeal pumping or response to touch, with physically damaged and bagged worms censored from analysis. (A) Ethosuximide had no effect on the lifespan (p > .05), (B) whereas MPS improved the longevity of the worms when compared to the DMSO control (p < .05). Mean lifespans (number of adulthood days) are as follows: succinimide, 12.45 ± 0.37 (n = 141); ethosuximide, 13.08 ± 0.39 (n = 139); DMSO, 11.51 ± 0.35 (n = 131); MPS, 14.37 ± 0.41 (n = 138). Data shown was pooled from three biological replicates, with comparisons of lifespans carried out with the log-rank test.

Notably, MPS and ethosuximide increased locomotion to a comparable extent despite a 160-fold difference in the external concentrations applied. As ageing is linked with the development of many neurodegenerative diseases, including ALS, the longevity effect of MPS on TDP-43(A315T)-expressing worms was also investigated. Despite the protective effects of 8 mM ethosuximide on locomotion, the drug did not significantly increase the shortened lifespan of these worms; whereas 0.05 mM MPS significantly increased mean lifespan by 25% (Fig. 6).

ALS is characterised by the degeneration of upper and lower motor neurons in the brain and spinal cord. To investigate if MPS has a direct protective effect against neuronal degeneration, the integrity of the GABAergic D-type inhibitory motor neurons was studied. These neurons innervate the body wall muscles of the nematode, and are required for ventral and dorsal muscle relaxation during locomotion. To visualise GABAergic neurons specifically, the TDP-43(A315T)-expressing strain was crossed with a transgenic strain containing GFP driven by the promoter for the *unc-25* gene, which encodes the GABA-synthesizing enzyme glutamic acid decarboxylase (Jin et al., 1999). Due to the transparency of *C. elegans*, the GFP-expressing GABAergic neurons can be visualised in vivo at various adult ages to track age-dependent neuronal changes. The cell bodies of the 19 GABAergic D-type inhibitory motor neurons lie along the ventral nerve cord and consist of 13 ventral (ventral D; VD) and 6 dorsal (dorsal D; DD) type neurons which innervate the respective ventral and dorsal body wall muscles of *C. elegans*.

**Fig. 7:**
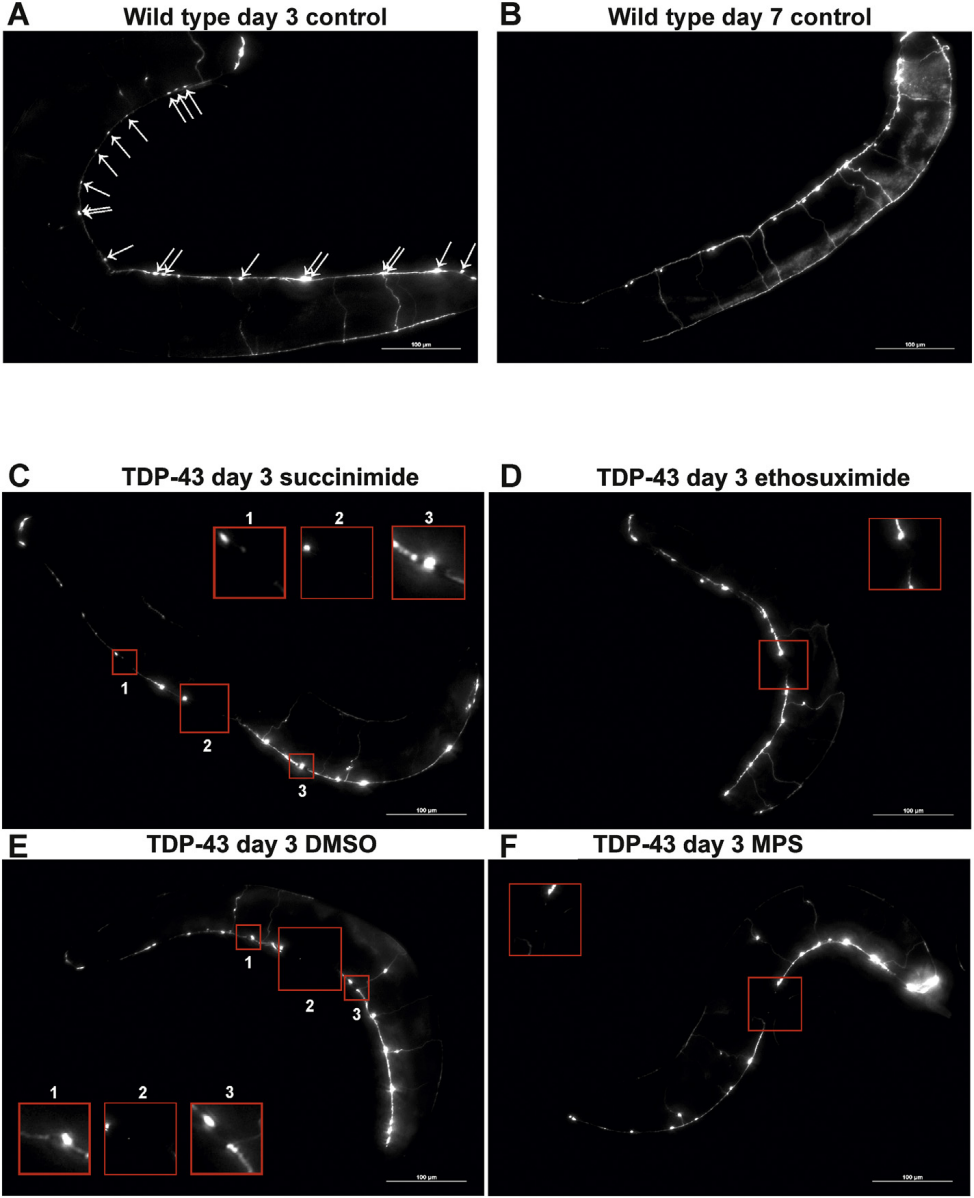
Representative images of worms expressing GFP in GABAergic neurons. Age-synchronised wild type worms expressing GFP under the control of the *unc-25* promoter are shown at adult ages of (A) day 3 and (B) day 7 to compare the appearance of D-type GABAergic motor neurons in the ventral nerve cord. The 19 cell bodies of this neuronal subset manifest as fluorescent punctae (indicated with arrows in A). (B) Ageing did not cause observable physical neuronal deterioration, which was evident from intact neuronal tracts and discernible cell bodies at adult age day 7. Images were taken at 200× magnification, scale bars = 100 μm. (C—F) Age-synchronised TDP-43(A315T)-expressing worms that also express GFP under the control of the *unc-25* promoter were chronically treated with (A) 8 mM succinimide, (B) 8 mM ethosuximide, (C) 0.4% DMSO vehicle or (D) 0.05 mM MPS in 0.4% DMSO from the L1 larval stage. Breaks within the D-type GABAergic motor neuron tracts in the ventral nerve cord are indicated by red boxes, which are magnified in the inset red boxes. Images were taken at 200× magnification, scale bars = 100 μm. (For interpretation of the references to colour in this figure legend, the reader is referred to the web version of this article.)

**Fig. 8:**
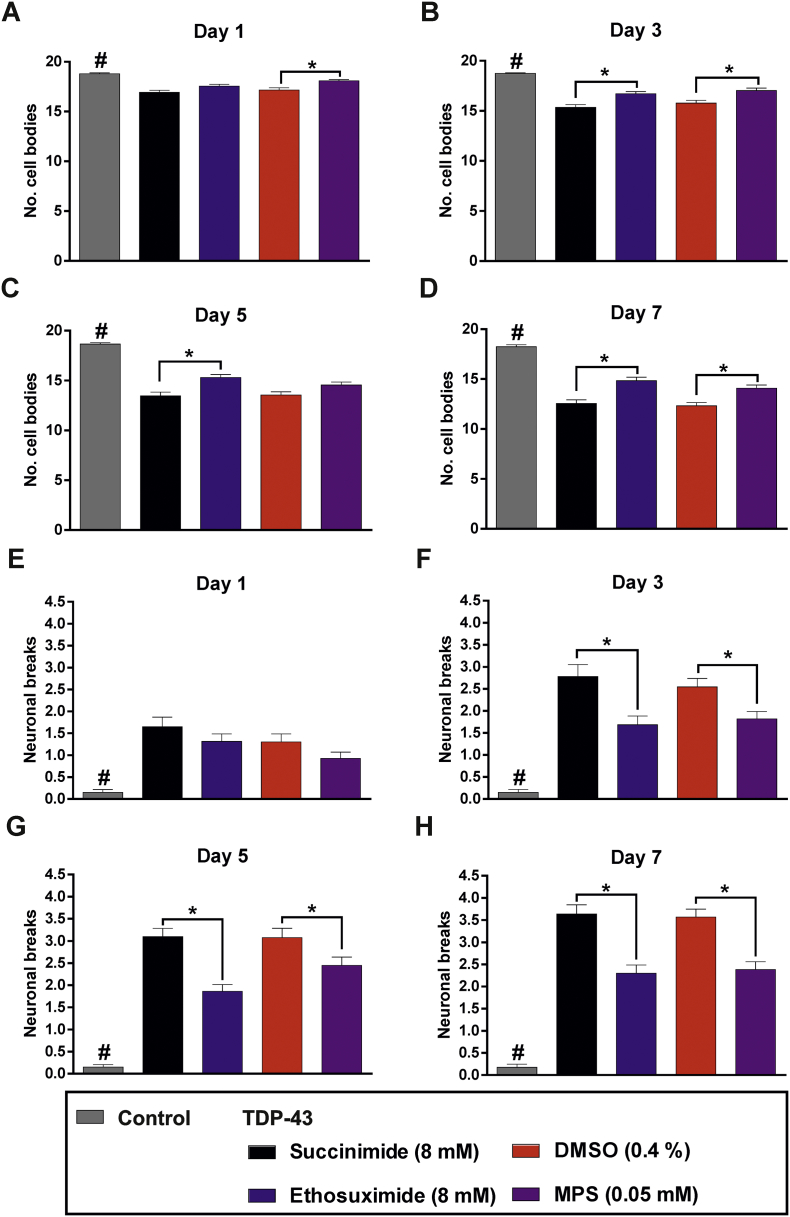
MPS ameliorates GABAergic neurodegeneration in the worm TDP-43 proteinopathy model. Age-synchronised wild type and TDP-43(A315T) worms expressing GFP under the control of the *unc-25* promoter were chronically treated with the indicated compounds from the L1 larval stage onwards. (A-D) Wild type worms consistently preserved more of the 19 D-type motor neuronal cell bodies than TDP43(A315T) expressing worms at all assayed ages, verifying neurodegenerative effect of the CK426 background (#, p < .05). Both ethosuximide (B-D) and MPS (A, B and D) protected against cell body losses in TDP-43 expressing worms when compared against respective succinimide and DMSO controls (p < .05). Data for wild type (n = 30 worms per age point) and TDP-43 strains (n = 33–47 worms per age point) were pooled from two and three biological replicates respectively. (E-H) Wild type control worms had fewer neuronal breaks than TDP-43 expressing worms at all ages, (#, p < .05). Both ethosuximide and MPS reduced the number of neuronal breaks in TDP-43 expressing worms from day 3 onwards as compared to succinimide and DMSO controls (p < .05). Data for wild type (n = 40 worms per age point) and TDP-43 expressing strains (n = 43–56 worms per age point) were pooled from three and four biological replicates respectively. All statistical comparisons in A-H were made via the Kruskal-Wallis test with Dunn's multiple comparisons; #, *, p < .05.

The integrity of this neuronal subset was assessed by quantifying the number of cell bodies and neuronal breaks in the ventral nerve cord, as previously described (Liachko et al., 2010b); cell bodies were evident from GFP punctae whereas discontinuities in the GFP fluorescence along the nerve cord highlight neuronal breaks. Control worms exhibited no significant neuronal damage even up to an adult age of day 7 (Fig. 7A,B). In contrast, TDP-43(A315T)-expressing worms suffered significant cell body losses and neuronal breaks from the youngest assessed age of day 1, verifying the neurodegenerative effect of the TDP-43 mutation (Fig. S2; Fig. S3; Fig. S4; Fig. 7C,E). Both ethosuximide and compound 9 conferred significant protection against cell body losses and breaks within this motor neuronal subset (Figs. 7B,D, 8), demonstrating that MPS ameliorates neurodegeneration with a similar efficacy to ethosuximide but with over 100-fold increased potency.

### 3.4 Determining compound bioaccumulation using ^1^H NMR

One simple explanation for MPS's greatly increased potency relative to ethosuximide could be that it bioaccumulates to higher levels within the worm than those externally applied. This seems unlikely, as for most compounds examined to date it has been shown that only a small fraction of the externally applied concentration actually permeates the worm's cuticle (Burns et al., 2010). Furthermore, the bioaccumulation scores calculated using the *C. elegans* bioaccumulation prediction algorithm (Burns et al., 2010) were very similar for ethosuximide and MPS (Table S2). Nevertheless, to test this directly, we set out to measure the bioaccumulation of applied compounds experimentally using ^1^H NMR spectroscopy (Fig. S5). Initial analysis of succinimide, ethosuximide and MPS generated reference spectra for each compound; succinimide was included for comparison to exclude poor bioaccumulation as a reason for its observed inactivity. Each compound yielded a unique pattern of characteristic ^1^H peaks deriving from their chemical structures (Fig. S6). For the best detectable signal-to-noise ratio from worm-derived metabolites, singlet peaks with the highest intensity for each compound were selected for further analysis and calibration curves were performed (Fig. S7) to enable subsequent quantification.

**Fig. 9:**
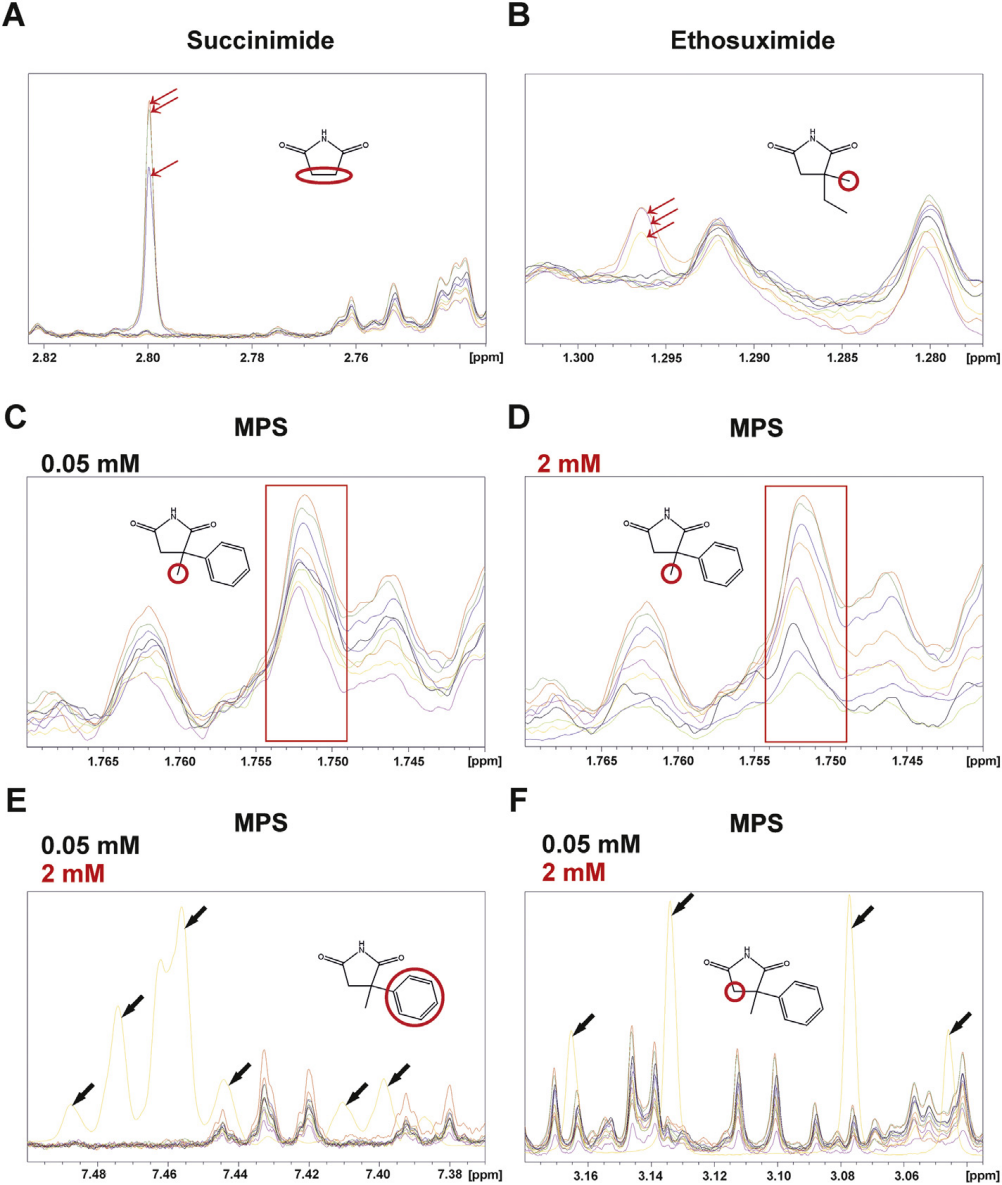
Determining compound bioaccumulation in TDP-43(A315T) worms model worms using ^1^H NMR. Overlay of spectra from lysates of chronically treated, age-synchronised day 1 adult TDP-43 expressing worms are shown, emphasising regions with compound-derived peaks of interest. (A) Succinimide (2.82–2.74 ppm) and (B) ethosuximide-derived peaks (1.30–1.28 ppm) were readily distinguishable in samples treated with an 8 mM external concentration of both compounds (red arrows). Peaks common to compound-treated and non-treated lysates arising from endogenous *C. elegans* metabolites are also shown alongside compound-derived peaks for comparison. Data shown are from nine ^1^H spectra from three treatment conditions with three biological replicates each. (For interpretation of the references to colour in this figure legend, the reader is referred to the web version of this article.) (C,D) The singlet peak with the highest intensity from MPS (red boxed region) was indistinguishable from endogenous metabolites in samples treated with either the optimal neuroprotective concentration of 0.05 mM (C) or the highest tolerable concentration of 2 mM (D). Due to the undetectability of the singlet peak, the presence of other compound-derived multiplet peaks in the aromatic (E) or –CH_2_ (F) region were analysed from spectra derived from treatment with both low and high concentrations. To facilitate peak identification from these multiplets, the reference spectrum of the pure compound was overlaid with those from treated worm lysates; reference peaks as shown in yellow and indicated with black arrows. These peaks were also not detectable from samples. Data shown is from nine ^1^H spectra from three treatment conditions with three biological replicates each.

TDP-43(A315T)-expressing worms chronically treated with succinimide, ethosuximide and MPS were lysed and extracted for ^1^H NMR analysis. A small fraction of each worm extract was processed in parallel using SDS-PAGE, to enable quantification of worm numbers in each sample by calculating total protein levels (Fig. S8). Following extraction and NMR analysis, the strongest singlet peaks from succinimide and ethosuximide were readily observable in worm lysates (Fig. 9A,B), whereas those from MPS were not detectable due to occlusion from *C. elegans* endogenous metabolites (Fig. 9C). However, the concentration of the externally administered MPS was 0.05 mM, which was 160-fold lower than the treatment concentration of 8 mM for both succinimide and ethosuximide. In an attempt to improve detectability of the MPS singlet peak, worms were administered a 40-fold higher concentration of the compound at 2 mM, the highest tolerable concentration determined from concentration-response assessments (Fig. 4). Nevertheless, this failed to distinguish the compound-9-derived signal from internal metabolites (Fig. 9D). Due to potential occlusion of this signal due to overlapping peaks from worm metabolites, other MPS-derived peaks occupying separate spectral regions were examined for both low and high concentrations of MPS. However, no distinguishable signals from the compound were visible at either concentration (Fig. 9E,F). The internal concentrations of succinimide and ethosuximide in TDP-43-expressing worms were quantified based on concentration and sample size standard curves (Fig. S7, Fig. S8). The measured internal concentrations were 206.6 ± 48.89 μM for succinimide and 143.8 ± 39.08 μM for ethosuximide, representing approximately 2.5% of the externally applied concentration. Although the lack of detectability of MPS prevents a precise estimate for its internal concentration, it seems reasonable to conclude that its greater potency compared to ethosuximide is not due to it bioaccumulating to high internal levels.

### 3.5 Mechanism of action of MPS

**Fig. 10:**
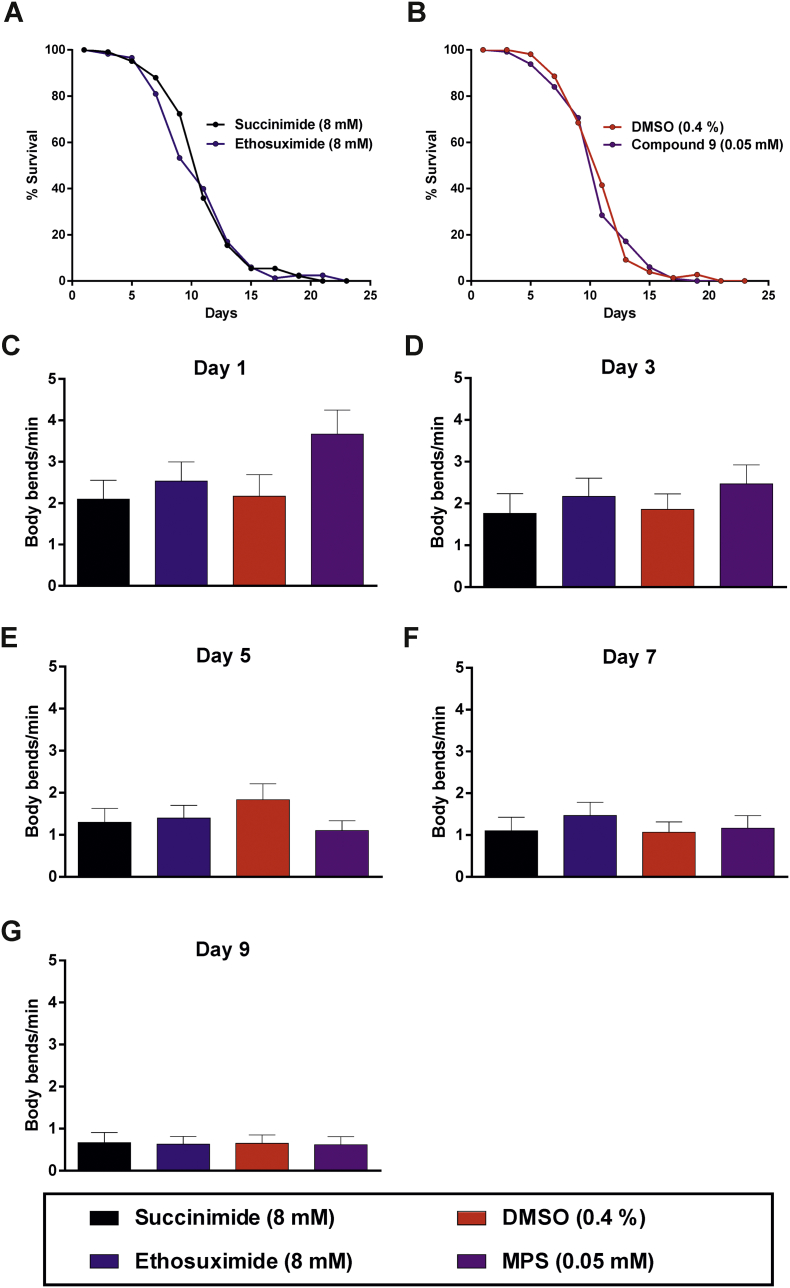
DAF-16 is essential for lifespan extension and neuroprotection by MPS. (A-B) Lifespan analyses were performed in chronically-treated *daf-16* null TDP-43(A315T) worms. Animals were scored as dead in the absence of pharyngeal pumping or response to touch, with physically damaged and bagged worms censored from analysis. Both ethosuximide (A) and MPS (B) failed to increase longevity in the absence of functional DAF-16 (p > .05). Mean lifespans (number of adulthood days) are as follows: succinimide, 11.70 ± 0.34 (n = 86); ethosuximide, 11.30 ± 0.33 (n = 87); DMSO, 12.03 ± 0.40 (n = 77); MPS, 11.65 ± 0.32 (n = 86). Data shown was pooled from two biological replicates, with comparisons of lifespans carried out with the log-rank test. (C-G) The ability of ethosuximide and MPS to mitigate the locomotion defects of TDP-43(A315T) worms was abolished by mutation of *daf-16*. Data was pooled from three biological replicates, with comparisons performed via the Kruskal-Wallis test with Dunn's multiple comparisons against the appropriate succinimide or DMSO controls (n = 26–30 worms per treatment per age point).

Having ruled out bioaccumulation, we reasoned that MPS's higher potency could be explained either by a greater ability to activate the same neuroprotective pathways as ethosuximide, or alternatively by activation of a different mechanism. We and others have shown that ethosuximide's neuroprotective and lifespan-extending effects in *C. elegans* are dependent on the FOXO transcription factor, DAF-16 (Tauffenberger et al., 2013; Chen et al., 2015b). Therefore, we introduced the null mutant *daf-16(mu86)* allele into our TDP-43(A315T)- and GABAergic-GFP-expressing strain by genetic crossing, in order to distinguish between these possibilities. The lifespan-extending effect of MPS was abolished in these *daf-16* mutant worms, as the mean lifespan of MPS-treated worms was reduced by 3% in comparison to DMSO controls (Fig. 10). Therefore, DAF-16 is essential for mediating the longevity effects of MPS. As predicted based on the lack of lifespan extension by ethosuximide described earlier (Fig. 6), the drug similarly exhibited no effect on the lifespan of these *daf-16* null worms (Fig. 10). Loss of DAF-16 function also abolished the neuroprotective effects of both compound 9 and ethosuximide as determined indirectly via locomotion (Fig. 10) and directly via GABAergic neurodegeneration (Figs. S9-S13). Therefore, we conclude that ethosuximide and MPS share a common downstream neuroprotective mechanism of action that requires DAF-16 transcription factor activity.

## 4 Discussion

Within the last 5 years, several different groups have independently reported neuroprotective effects of the established anti-epileptic drug ethosuximide in a variety of neurodegenerative disease models. These range from *C. elegans* models of ALS (Tauffenberger et al., 2013), frontotemporal dementia with parkinsonism-17 (Chen et al., 2015b) and adult-onset neuronal ceroid lipofuscinosis (Chen et al., 2015b)); a mammalian cell culture model of Huntington's disease (Chen et al., 2015b); and a rat in vivo amyloid beta toxin-induced AD model (Tiwari et al., 2015). Clearly, ethosuximide holds promise for repurposing as a potential new treatment for neurodegenerative diseases, but the high concentrations required for its effects and an unknown molecular mechanism of neuroprotective action represent significant challenges. Here we have used a combination of chemoinformatics and phenotypic screening using *C. elegans* to identify a novel neuroprotective activity of the ethosuximide-related compound, α-methyl-α-phenylsuccinimide (MPS). Importantly, MPS is as effective as ethosuximide in ameliorating neurodegeneration despite being applied at a 160-fold lower external concentration. MPS was undetectable by NMR in worm extracts, likely due to occlusion of the expected peak regions by endogenous metabolites. Alternatively, it could be that MPS was further metabolized into another compound that may itself possess bioactivity. The only reported metabolites of MPS are its para-hydroxylation product, α-(*p*-hydroxyphenyl)-α-methylsuccinimide, and a glucuronidate-conjugated form of this molecule (Dudley et al., 1974). However, we were unable to detect the predicted NMR signals corresponding specifically to these reported metabolites, or indeed for any other potential MPS-derived signals in NMR spectra using metabolomic analysis. Hence, MPS and any putative metabolite(s) are evidently undetectable by NMR, presumably due to rapid clearance and/or occlusion of peaks by strong signals from endogenous worm metabolites. This therefore precludes a precise value for MPS's effective therapeutic concentration within the worm. Nevertheless, as the calculated *C. elegans* bioaccumulation scores for ethosuximide and MPS are similar; and as we and others (Evason et al., 2005) have shown that only 1–5% of external applied ethosuximide bioaccumulates inside worms; it seems reasonable to assume that the optimal externally applied concentration of 0.05 mM MPS corresponds to a sub-micromolar internal concentration inside the worm. This should facilitate future identification of the direct molecular targets of MPS using affinity chromatography/proteomic approaches, which has recently been optimised to identify the protein targets of various small molecules with low micromolar binding affinities (Ong et al., 2009). Furthermore, MPS provides more flexibility than ethosuximide for making chemical modifications to enable linkage to affinity matrices, by virtue of its phenyl moiety. Given that we have shown here that ethosuximide and MPS share a common downstream mechanism of action requiring the DAF-16 transcription factor, it seems likely that such studies would also shed light on ethosuximide's currently unclear molecular mechanisms of action in both epilepsy and neuroprotection. Interestingly, several other neuroprotective compounds are also dependent on DAF-16, including steroids, polyphenols, lithium and metformin, suggesting that a comparative network analysis of these drugs and their targets could also shed light on the molecular mechanism of action of MPS and ethosuximide (Chen et al., 2015a; Farina et al., 2017). In addition, the ease of chemical modification of MPS will also facilitate future medicinal chemistry studies to find even more potent 2nd-generation succinimide-based neuroprotective agents. MPS's high MPO scores indicating likely good CNS penetration after oral delivery suggest that the MPS scaffold is a suitable starting point for such medicinal chemistry optimisation.

MPS is the active metabolite of the existing clinically approved AED, methsuximide. Indeed, methsuximide is a pro-drug that is demethylated in vivo to yield *N*-desmethylmethsuximide, which is identical to MPS and is responsible for its antiepileptic effects in patients (Strong et al., 1974). Methsuximide may therefore have translational potential for repurposing to treat TDP-43 proteinopathies. Interestingly, methsuximide is effective against a variety of different forms of epilepsy, including complex partial seizures (Wilder and Buchanan, 1981; Browne et al., 1983), intractable childhood seizures (Tennison et al., 1991; Sigler et al., 2001) and juvenile myoclonic epilepsy (Hurst, 1996); whereas ethosuximide is only effective against absence seizures (Goren and Onat, 2007). These differences are also seen in rodents, where ethosuximide is effective solely in the PTZ seizure model whereas methsuximide also prevents maximal electroshock-induced seizures (Chen et al., 1963). This may suggest that the two drugs have different therapeutic mechanisms of action in epilepsy; or alternatively that ethosuximide can prevent convulsive seizures, but only at high doses that induce toxicity in patients. Our findings here that ethosuximide and MPS share a common downstream DAF-16-dependent mechanism of neuroprotective action support the latter hypothesis; as does the observation that ethosuximide is capable of preventing maximal electroshock-induced seizures in mice, albeit at an ED_50_ that is 20-fold higher than methsuximide and is accompanied by anaesthetic effects (Chen et al., 1963). High doses of the methsuximide pro-drug have been associated with toxicity due to serum MPS levels of over 0.2 mM (Sigler et al., 2001), which could hinder its therapeutic use in neurodegenerative disease. However, given that we have shown here that MPS ameliorates neurodegeneration in *C. elegans* at a much lower concentration than this, it may be that methsuximide would have neuroprotective activity at lower doses than used for treatment of epilepsy, which may avoid such adverse effects. Once converted from the pro-drug methsuximide, MPS has a long half-life of 38–70 h in humans (Strong et al., 1974; Porter et al., 1979), so relatively infrequent low oral doses of methsuximide could potentially provide long-lasting neuroprotection.

## 5 Conclusion

MPS is a novel neuroprotective molecule that is more than two orders of magnitude more potent than the related molecule, ethosuximide. Its increased potency will aid future efforts to identify the currently unknown direct molecular targets of both MPS and ethosuximide, given that they share a common downstream neuroprotective mechanism of action. As MPS is the active metabolite of the antiepileptic pro-drug, methsuximide, treatment with low doses of methsuximide could potentially be considered as a treatment for TDP-43 proteinopathies such as amyotrophic lateral sclerosis and FTLD-TDP, and possibly other human neurodegenerative diseases.

The following are the supplementary data related to this article.

Supplementary Video 1 Convulsions in worms treated with inactive succinimide. Supplementary Video 1

Supplementary Video 2 Rescue of convulsions in worms treated with ethosuximide. Supplementary Video 2

Supplementary material Image 1

## Ethics approval and consent to participate

Not applicable.

## Consent for publication

Not applicable.

## Availability of data and material

Not applicable.

## Competing interests

The authors declare no competing interests.

## Funding

SW was supported by a Wellcome Trust PhD studentship awarded to AM and RDB (grant number 102378/Z/13). BCK was supported by the US department of Veterans Affairs (Merit Review grants # I01BX003755 and I01BX002619). NMR work was part-funded by an award from the University of Liverpool Technology Directorate Voucher Scheme (www.liverpool.ac.uk/technology-directorate) to AM. Software licences for data analysis used in the Shared Research Facility for NMR metabolomics are funded by the MRC Clinical Research Capabilities and Technologies Initiative (MR/M009114/1).

## Author contributions

SW performed studies involving chemistry, *C. elegans* and NMR techniques, analysed and interpreted data, and drafted the manuscript. MGP performed *C. elegans* experiments and analysed data. MMP designed and assisted with NMR studies, and analysed and interpreted NMR data. CP designed and performed organic compound synthesis. BCK provided essential reagents and materials. JWB helped design *C. elegans* experiments and analysed data. NGB conceived, designed and assisted with the chemoinformatic studies and analysed data. PON conceived and designed the chemoinformatic studies, designed organic compound synthesis and analysed data. RDB conceived of the study, participated in its design and coordination, and analysed data. AM conceived of the study, designed and coordinated experiments, analysed data and drafted the manuscript with input from all of the other authors.
