# Supplementary material for: α-Methyl-α-phenylsuccinimide ameliorates neurodegeneration in a C. elegans model of TDP-43 proteinopathy
Source: Neurobiol Dis. 2018 Oct;118:40–54. doi: 10.1016/j.nbd.2018.06.013 (PMC6097874; doi:10.1016/j.nbd.2018.06.013)
Supplement: Supplementary file 3 — Supplementary material [file mmc3.pdf]

Table S1. CNS MPO scores of selected compounds.

No.	Name	SMILES	CLogP		CLogD		TPSA		MW		HBD		pKa		**CNS MPO Score
			Actual	*T0	Actual	*T0	Actual	*T0	Actual	*T0	Actual	*T0	Actual	*T0	
1	Succinimide	C1CC(=O)NC1=O	-1.17	1	-0.67	1	46.17	1	99.09	1	1	0.83	9.62	0.19	5.0
2	Ethosuximide	CCC1(CC(=O)NC1=O)C	0.39	1	0.40	1	46.17	1	141.17	1	1	0.83	9.70	0.15	5.0
†3	5,5-Diphenylhydantoin sodium salt	C1=CC=C(C=C1)C2(C(=O)[N-]C(=O)N2)C3=CC=CC=C3.[Na+]	2.09	1	2.47	0.77	58.2	1	252.27	1	2	0.5	14.81	0	4.3
4	Trimethadione	CC1(C(=O)N(C(=O)O1)C)C	-0.12	1	-0.06	1	46.61	1	143.14	1	0	1	-2.18	1	6.0
5	Thalidomide	C1CC(=O)NC(=O)C1N2C(=O)C3=CC=CC=C3C2=O	0.53	1	0.53	1	83.55	1	258.23	1	1	0.83	10.70	0	4.8
6	Phthalimide	C1=CC=C2C(=C1)C(=O)NC2=O	1.15	1	1.1	1	46.17	1	147.13	1	1	0.83	10.39	0	4.8
7	2-Pyrrolidone	C1CC(=O)NC1	-0.97	1	-0.64	1	29.1	0.46	85.11	1	1	0.83	16.62	0	4.3
8	N-Phenylphthalimide	C1=CC=C(C=C1)N2C(=O)C3=CC=CC=C3C2=O	2.39	1	2.33	0.84	37.38	0.87	223.23	1	0	1	-0.54	1	5.7
9	α-Methyl-α-phenylsuccinimide	CC1(CC(=O)NC1=O)C2=CC=CC=C2	0.91	1	1.36	1	46.17	1	189.21	1	1	0.83	9.17	0.42	5.2
10	1-Benzylpyrrolidine-2,5-dione	C1CC(=O)N(C1=O)CC2=CC=CC=C2	0.97	1	1.43	1	37.38	0.87	189.21	1	0	1	-1.37	1	5.9
11	α-Methyl-α-propylsuccinimide	CCCC1(CC(=O)NC1=O)C	0.92	1	0.91	1	46.17	1	155.20	1	1	0.83	9.70	0.15	5.0
12	3,3-Diethylpyrrolidine-2,5-dione	CCC1(CC(=O)NC1=O)CC	0.92	1	1.25	1	46.17	1	155.20	1	1	0.83	9.70	0.15	5.0
13	4-Ethyl-4-methylpyrrolidin-2-one	CCC1(CC(=O)NC1)C	0.59	1	1.31	1	29.10	0.46	127.19	1	1	0.83	16.70	0	4.3
14	α,α-Dimethyl-β-methylsuccinimide	CC1C(=O)NC(=O)C1(C)C	0.39	1	0.3	1	46.17	1	141.17	1	1	0.83	9.74	0.13	5.0
15	3,3-Diethylpyrrolidin-2-one	CCC1(CCNC1=O)CC	1.12	1	0.86	1	29.10	0.46	141.21	1	1	0.83	16.70	0	4.3
16	Methsuximide	CC1(CC(=O)N(C1=O)C)C2=CC=CC=C2	1.46	1	1.67	1	37.38	0.87	203.24	1	0	1	-1.81	1	5.9
17	Phensuximide	CN1C(=O)CC(C1=O)C2=CC=CC=C2	0.94	1	1.68	1	37.38	0.87	189.21	1	0	1	-1.86	1	5.9
18	2,5-Pyrrolidinedione,3-ethyl-1,3-dimethyl	CCC1(CC(=O)N(C1=O)C)C	0.95	1	0.75	1	37.38	0.87	155.19	1	0	1	-1.25	1	5.9

The CNS MPO algorithm predicts the CNS permeability of compounds from the following six key physicochemical parameters commonly considered by medicinal chemists in drug design and CNS penetration: calculated partition (ClogP) and dissociation (ClogD) coefficients, molecular weight (MW), topological polar surface area (TPSA), number of hydrogen bond donors (HBD), and dissociation constant (pK_a). Actual values of these parameters are transformed (T0) and summation of the six T0 scores (*) yields the final CNS MPO score (**), which gives a numerical measure of predicted CNS permeation; the greater the score is, up to a maximum of 6, the better the predicted permeability. †The CNS MPO score for the phenytoin sodium salt (compound 3) was derived based on physicochemical properties for phenytoin due to the difficulty for *in silico* modelling of the salt.

Table S2. Rankings of screened compounds.

Compound	No.	Solubility (LogS)	CNS MPO score	Similarity (Tc value)	Predicted *C. elegans* bioaccumulation score	Pareto ranking
Trimethadione	4	-0.534	6	0.208	-0.339	1
2,5-Pyrrolidinedione,3-ethyl-1,3-dimethyl	18	-1.056	5.9	0.304	-0.804	1
1–Benzylpyrrolidine-2,5-dione	10	-1.79	5.9	0.143	-0.0670	1
Ethosuximide	2	-1.11	5	1	-0.203	1
Phthalimide	6	-1.44	4.8	0.261	1.66	1
3,3-Diethylpyrrolidin-2-one	15	-1.71	4.3	0.550	0.154	1
α-Methyl-α-phenylsuccinimide	9	-2.43	5.2	0.400	0.322	1
3,3-Diethylpyrrolidine-2,5-dione	12	-1.46	5	0.875	-0.127	1
N-Phenylphthalimide	8	-3.03	5.7	0.148	0.0538	1
Methsuximide	16	-2.39	5.9	0.172	-0.279	1
Phensuximide	17	-1.95	5.9	0.13333	-1.06	2
α-Methyl-α-propylsuccinimide	11	-1.53	5	0.824	-1.10	2
5,5-Diphenylhydantoin sodium salt	3	-3.53	4.3	0.222	1.10	2
4-Ethyl-4-methylpyrrolidin-2-one	13	-1.33	4.3	0.632	-0.350	2
Succinimide	1	0.0350	5	0.563	-0.643	2
α,α-Dimethyl-β-methylsuccinimide	14	-1.16	5	0.526	-1.29	3
2-Pyrrolidine	7	-0.0560	4.3	0.400	-0.666	3
Thalidomide	5	-2.11	4.8	0.300	-1.69	4

Compounds were ranked on Pipeline Pilot™ via the “Pareto Sort” function, based on structural similarity to ethosuximide, and CNS MPO and *C. elegans* bioaccumulation scores. Predicted aqueous solubility (LogS) values are also indicated.

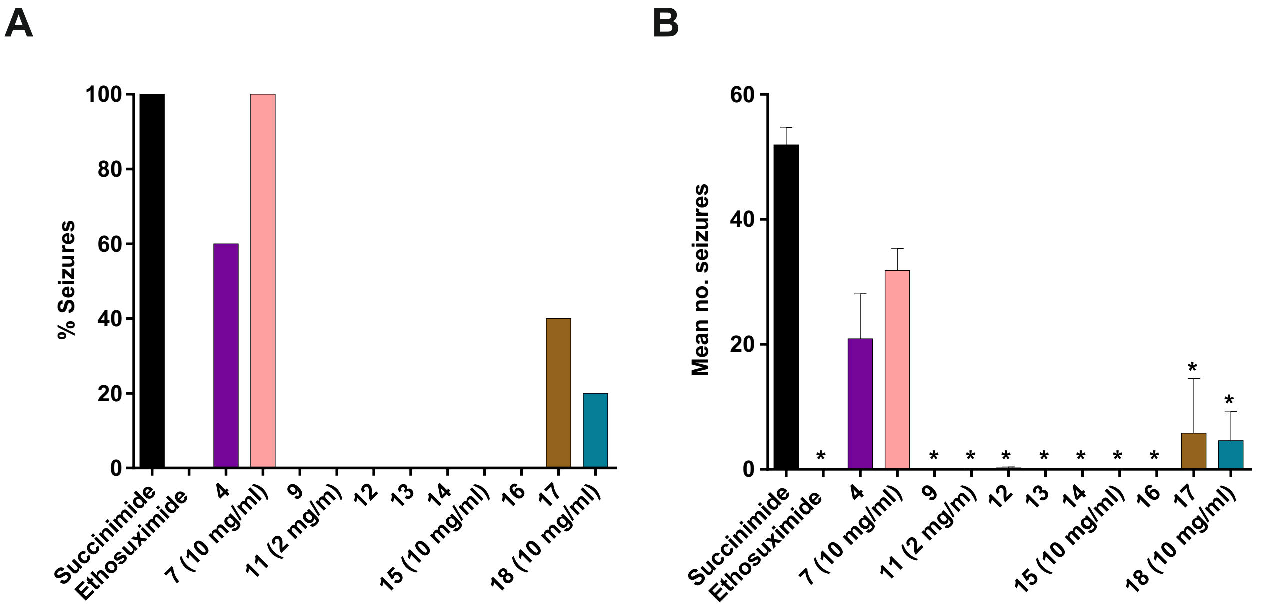

Figure S1. Preliminary compound screen for bioactivity in a *C. elegans* PTZ-induced seizure assay. Unless indicated otherwise, screening was performed at the optimal anticonvulsive concentration of ethosuximide at 4 mg/ml. Compounds were assessed at a lower concentration of 2 mg/ml in the event of evident toxicity, or at a higher concentration of 10 mg/ml to verify inactivity if observed at 4 mg/ml. With the exception of compounds 4 and 7, all compounds reduced both (A) percentage of seizing worms and (B) frequency of seizures ($p<0.05$). Compound 4 was not consistently active but displayed anticonvulsive protection in some worms, therefore it was herein classified as partially active. Data shown was pooled from up to two biological replicates, with comparison of seizure rates carried out using the Kruskal-Wallis test with Dunn's multiple comparison (n = 5 – 20 worms per compound); *, $p<0.05$.

Table S3. Structural features and potencies of screened compounds.

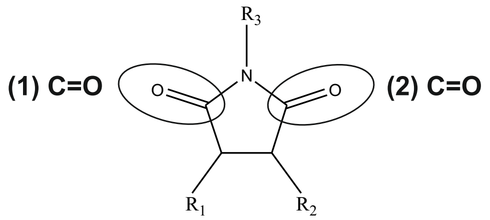

Compound	No.	Structure	R_1	R_2	R_3	(1) C=O	(2) C=O	Active?	EC_{50} (mM)	Potency against ethosuximide
**Ethosuximide**	**2**	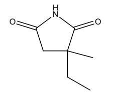	**X**	✓	**X**	✓	✓	**ACTIVE**	**9.7**	**-**
Succinimide	1	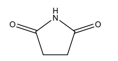	X	X	X	✓	✓	**Inactive**	-	-
2-Pyrrolidine	7	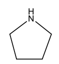	X	X	X	✓	X		-	-
Trimethadione	4	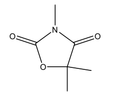	X	✓	✓	✓	✓	**Active but no differences in potency or less potent than ethosuximide**	29.2	3-fold lower
α-Methyl-α-propylsuccinimide	11	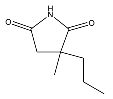	X	✓	X	✓	✓		9.7	No significant difference
3,3-Diethylpyrrolidine-2,5-dione	12	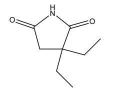	X	✓	X	✓	✓		12.4	No significant difference
4-Ethyl-4-methylpyrrolidin-2-one	13	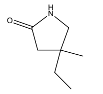	X	✓	X	✓	X		16.5	2-fold lower
3,3-Diethylpyrrolidin-2-one	15	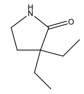	X	✓	X	X	✓		41.6	*** 3-fold lower than Compound 12**
Phensuximide	17	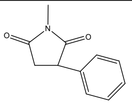	X	✓	✓	✓	✓		9.9	No significant difference
2,5-Pyrrolidinedione, 3-ethyl-1,3-dimethyl	18	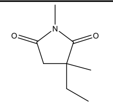	X	✓	✓	✓	✓		29.3	3-fold lower
α-Methyl-α-phenylsuccinimide	9	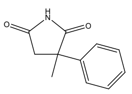	X	✓	X	✓	✓	**Active and more potent than ethosuxmimide**	4.6	2-fold higher
α,α-Dimethyl-β-methylsuccinimide	14	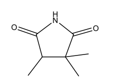	✓	✓	X	✓	✓		4.7	2-fold higher
Methsuximide	16	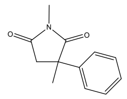	X	✓	✓	✓	✓		4.1	2-fold higher

Potencies (Ec_{50} values) were compared relative to ethosuximide (in bold), with the exception of compound 15 whiich was compared against compound 12 due to a common R_2 diethyl moiety whereas ethosuximide has a methyl-ethyl one.

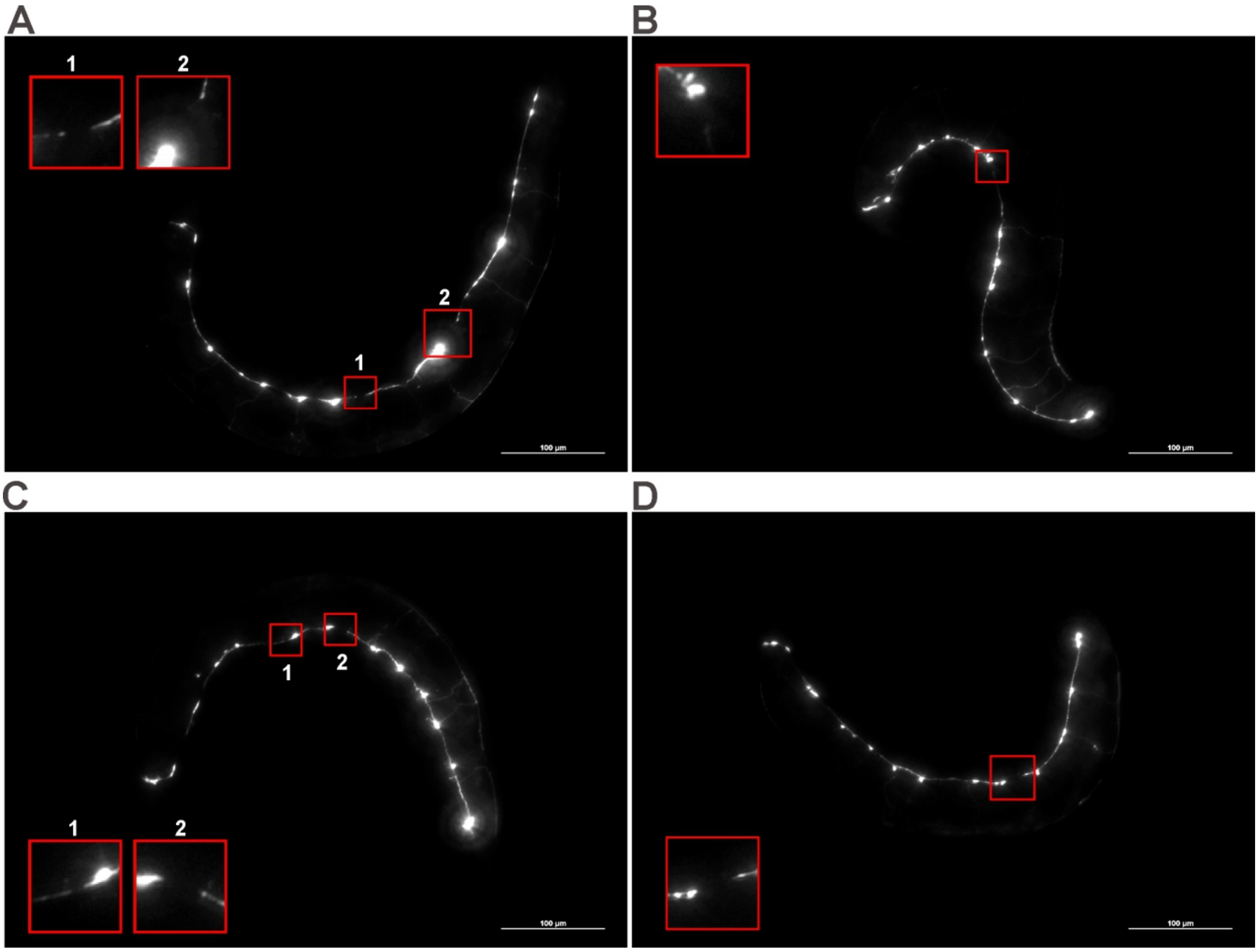

Figure S2. Representative images of day 1 TDP-43(A315T) worms expressing GFP in GABAergic neurons. Age-synchronised worms were chronically treated with 8mM (A) succinimide or (B) ethosuximide, (C) 0.4 % DMSO vehicle or (D) 0.05 mM MPS in 0.4 % DMSO from the L1 larval stage. Breaks within the D-type GABAergic motor neurons in the ventral nerve cord are indicated and enlarged with insets (red boxes). Images were acquired at 200X magnification, scale bars = 100 µm.

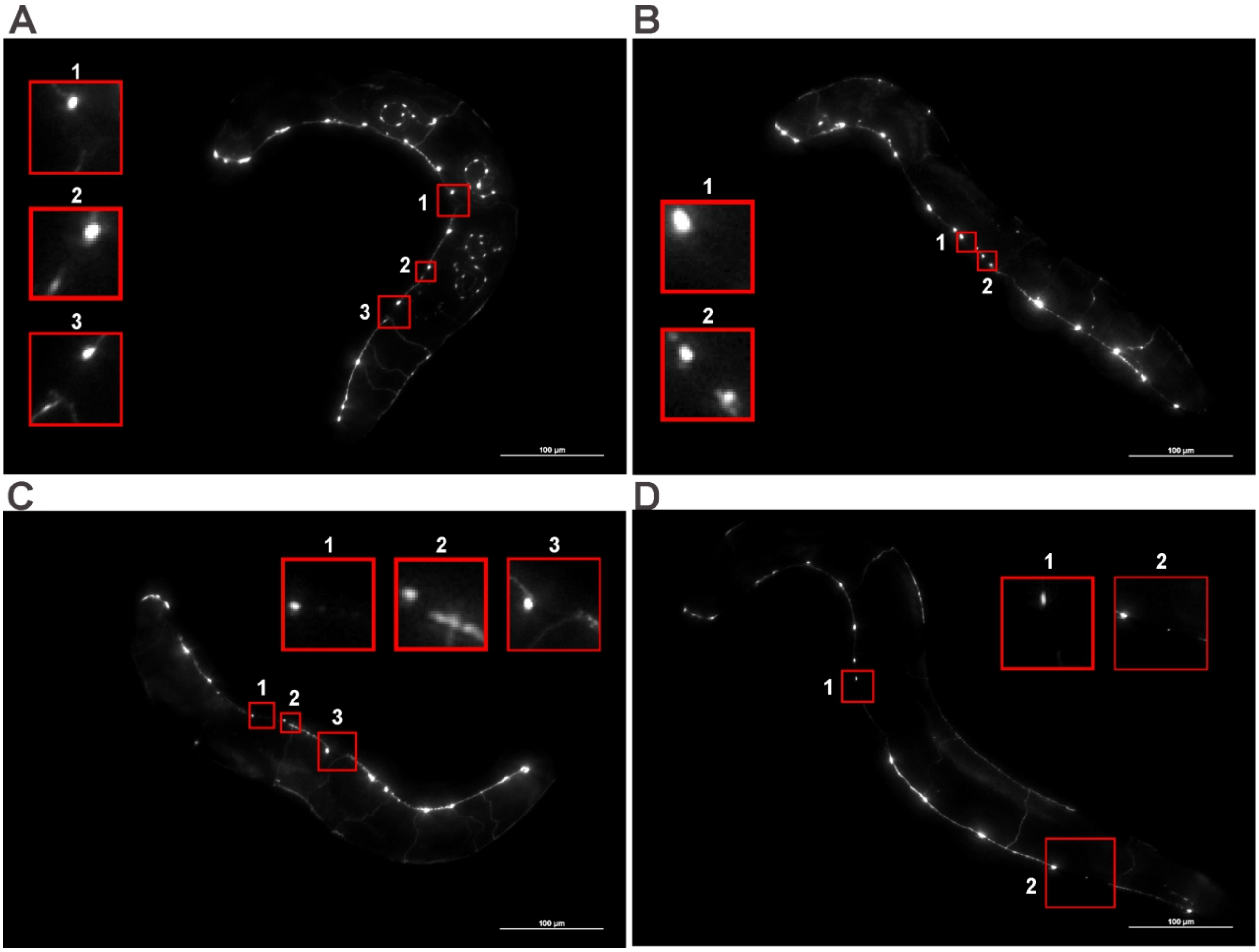

Figure S3. Representative images of day 5 TDP-43(A315T) worms expressing GFP in GABAergic neurons. Age-synchronised worms were chronically treated with 8mM (A) succinimide or (B) ethosuximide, (C) 0.4 % DMSO vehicle or (D) 0.05 mM MPS in 0.4 % DMSO from the L1 larval stage. Breaks within the D-type GABAergic motor neurons in the ventral nerve cord are indicated and enlarged with insets (red boxes). Bagged worms, which contain hatched progenies as shown by the representative image for (A) succinimide-treated worms, were commonly observed at age day 5. Images were taken at 200X magnification, scale bars = 100 µm.

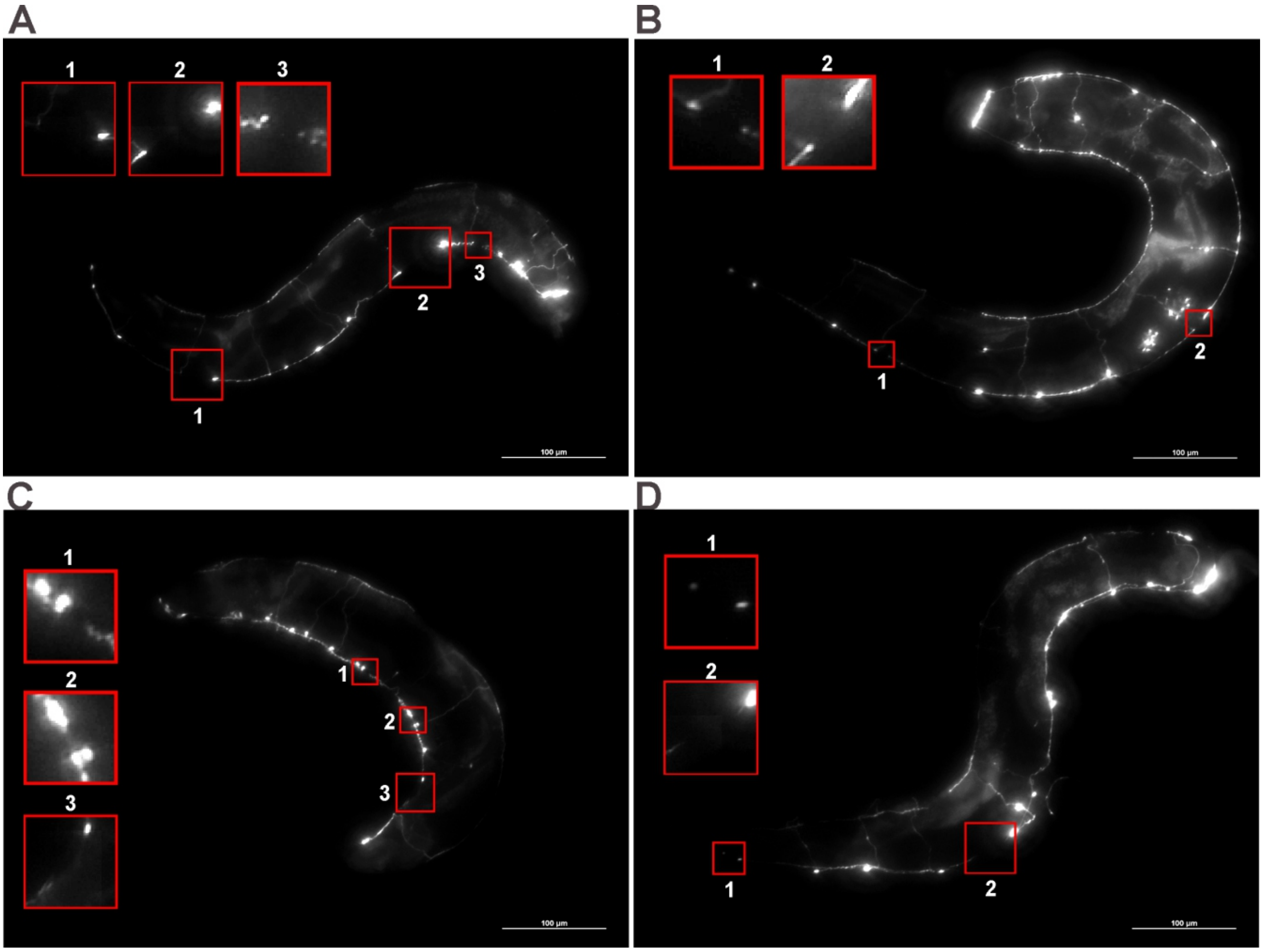

Figure S4. Representative images of day 7 TDP-43(A315T) worms expressing GFP in GABAergic neurons. Age-synchronised worms were chronically treated with 8mM (A) succinimide or (B) ethosuximide, (C) 0.4 % DMSO vehicle or (D) 0.05 mM MPS in 0.4 % DMSO from the L1 larval stage. Breaks within the D-type GABAergic motor neurons in the ventral nerve cord are indicated and enlarged with insets (red boxes). Bagged worms were also commonly observed at this age. Images were taken at 200X magnification, scale bars =

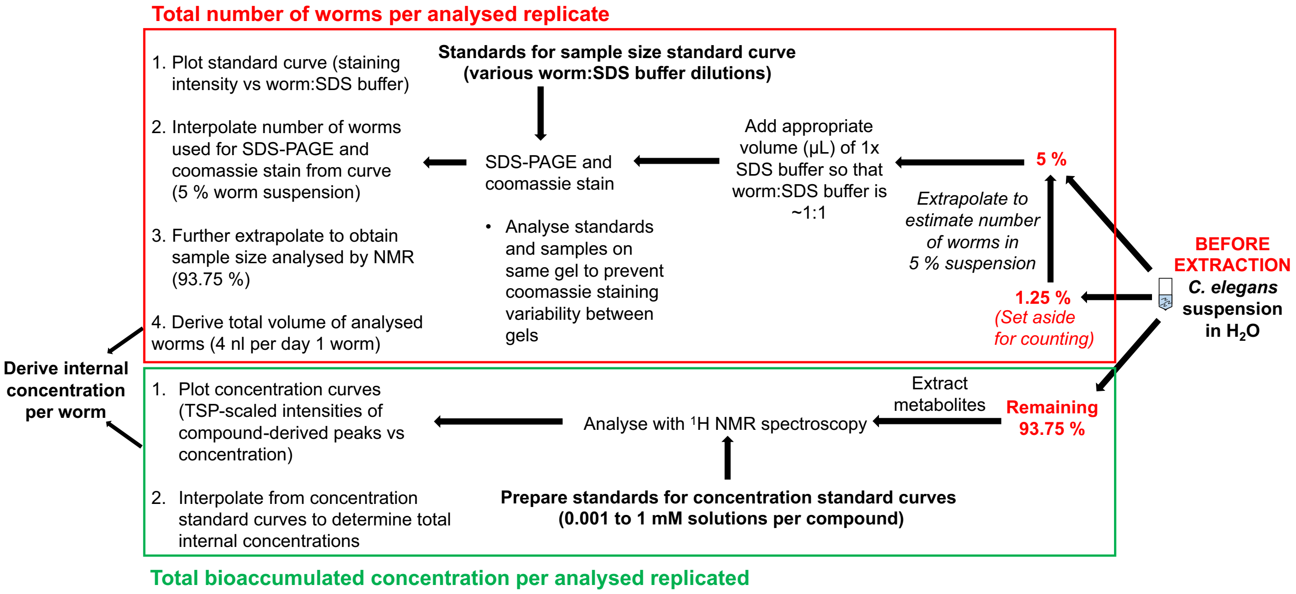

Figure S5. Workflow for determining bioaccumulated drug concentration in day 1 TDP-43(A315T)-expressing *C. elegans*.

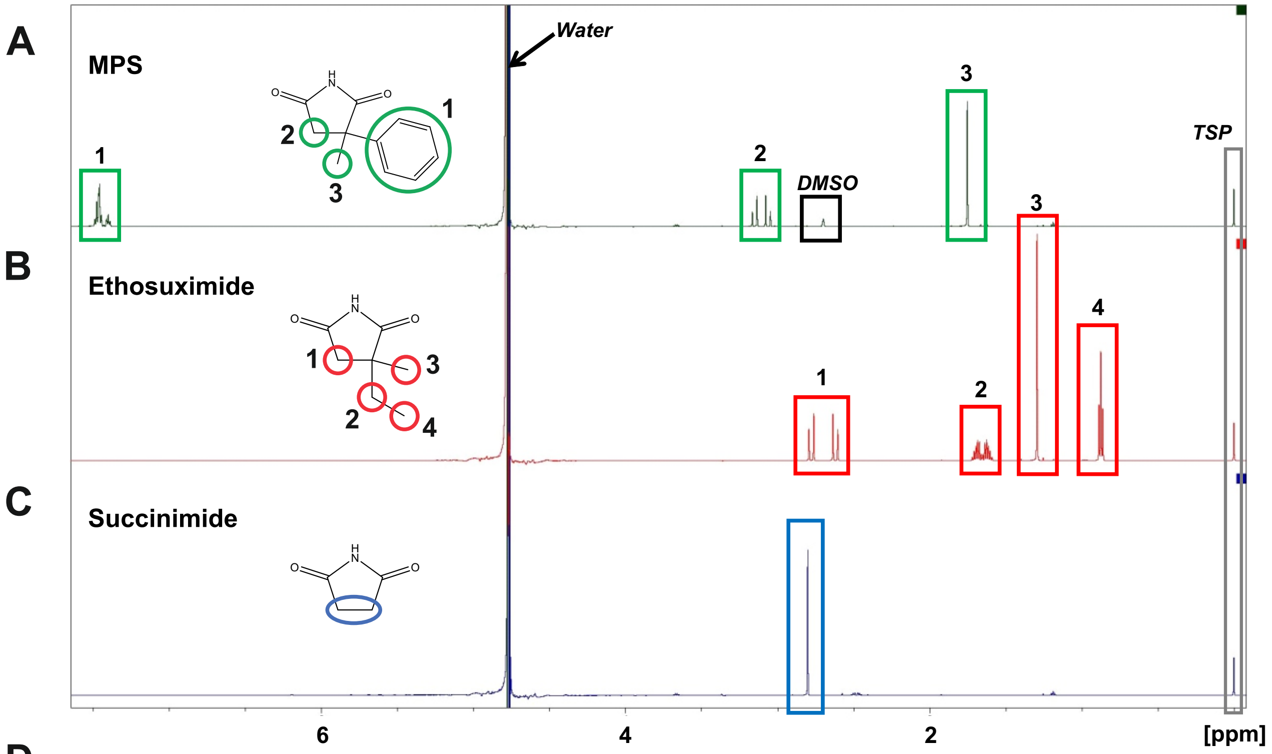

D

Compound	Chemical group no.	^{1}H Chemical shift (ppm)
Succinimide	-	2.820 – 2.787
Ethosuximide	1	2.798 - 2.593
	2	1.730 - 1.580
	3	1.299 – 1.294
	4	0.90 - 0.85
MPS	1	7.50 - 7.37
	2	3.18 - 3.03
	3	1.758 -1.746
DMSO	-	2.743 – 2.730

Figure S6. Compound-derived 1D ^{1}H NMR spectra. One mM solutions of compounds (A, MPS; B, ethosuximide; C, succinimide) were analysed with 1D ^{1}H NMR spectroscopy. Chemical groups from each compound yielded a distinct pattern of peaks at various chemical shifts, which enables the differentiation of one compound from another; groups and corresponding peaks are numbered. Note that the DMSO-derived peak from the MPS-derived spectrum was from the residual 1 % protonated component of a 99 % deuterated form of the vehicle used for solubilising the compound. Peaks from protonated water and the reference compound TSP are also indicated. (D) Table indicating spectral regions at which peaks appear. Peaks with the highest intensities were selected for future analysis (peaks number 3 for ethosuximide and MPS, and singlet peak for succinimide).

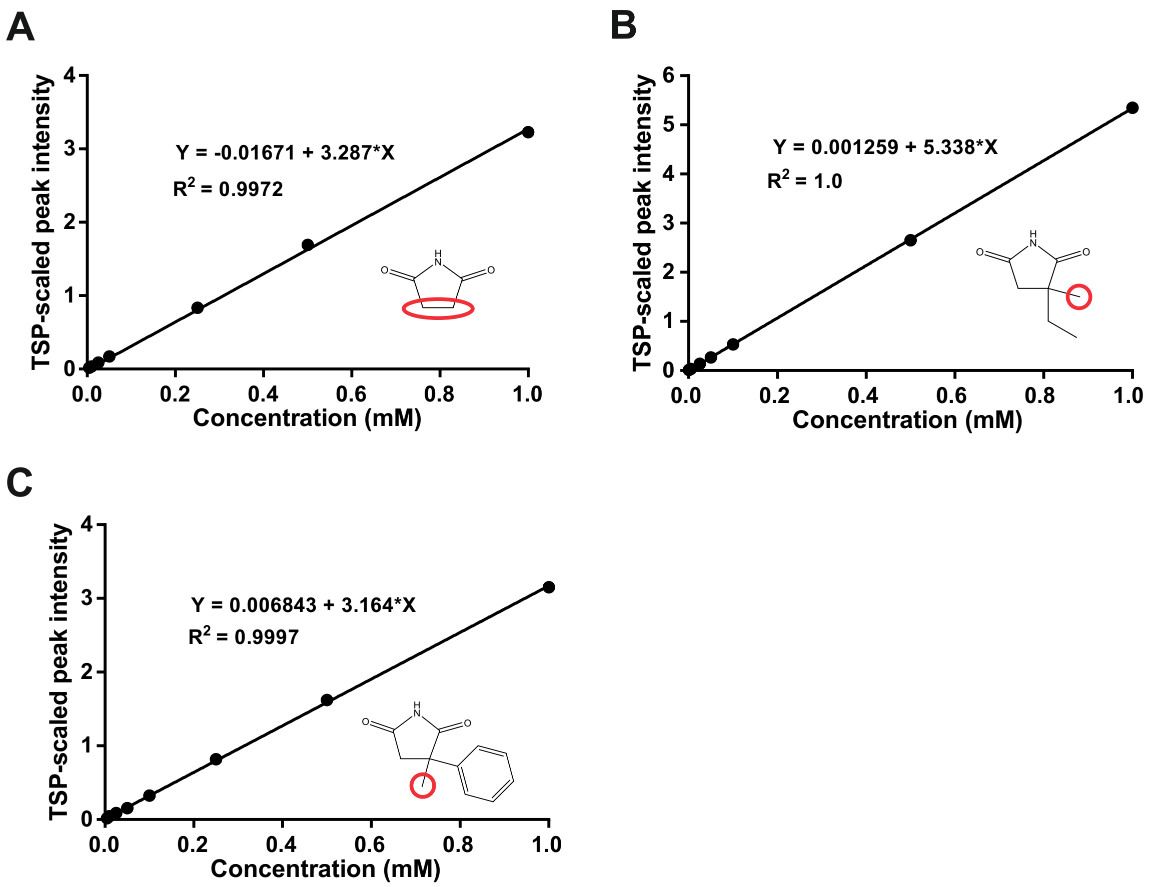

Figure S7. Standard curves for estimating internal concentration of compounds in *C. elegans* following treatment. A range of concentrations for each compound (A, succinimide, B, ethosuximide, C, MPS) were analysed with 1D ^{1}H NMR spectroscopy and the resultant spectral intensities of analysed peaks, as derived from indicated regions from each compound, were scaled to the reference compound TSP and plotted against respective concentrations to generate the curves.

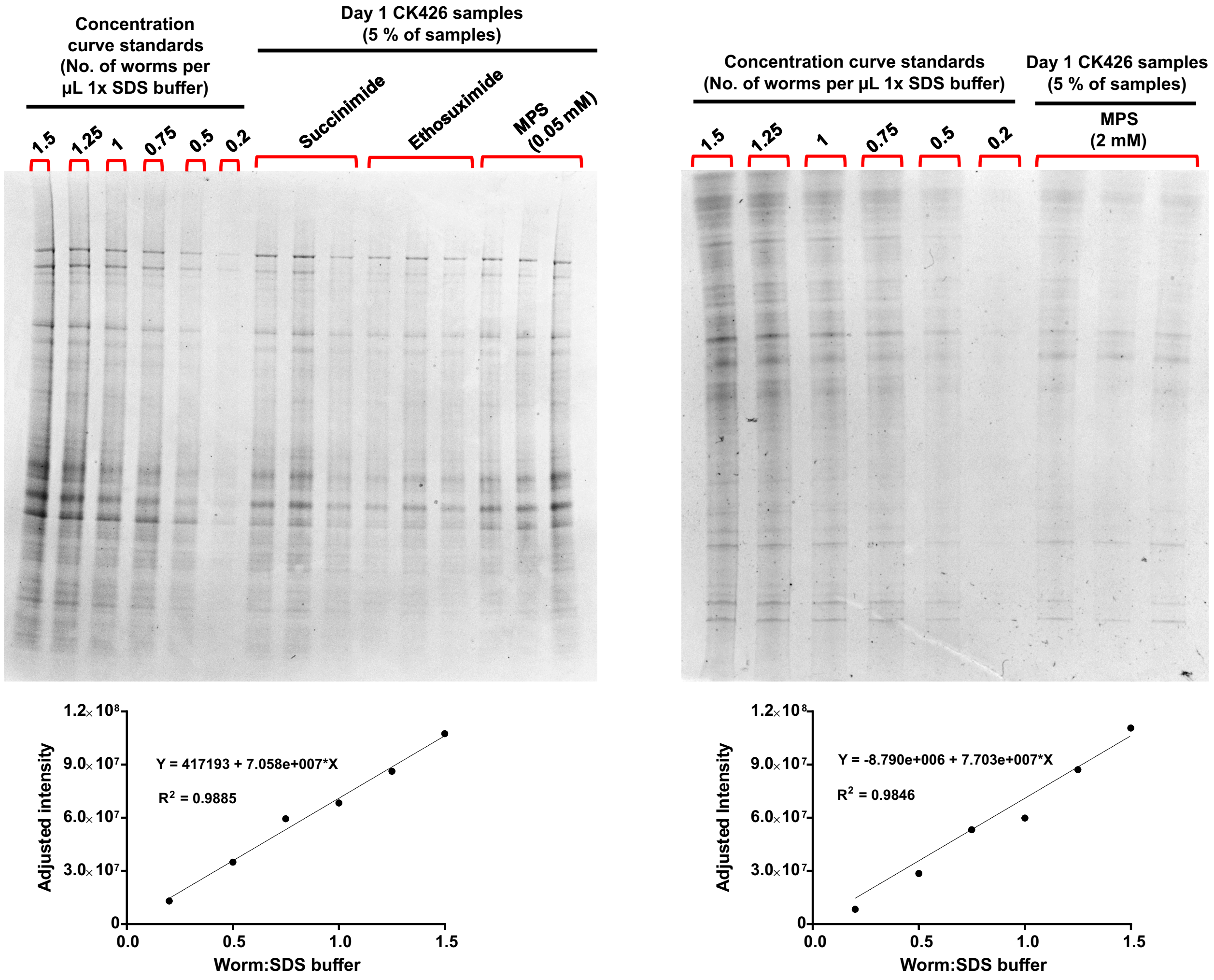

Figure S8. Sample size estimation from total protein standard curves. Standard curves were generated from the coomassie blue staining intensities of known ratios of day 1 TDP-43(A315T) worms in 1X SDS buffer resolved on SDS-PAGE gel. Samples, which are 5 % of the analysed populations, were resolved and stained alongside standards to preserve staining consistency for accurate interpolation. Shown here are the stained gels and corresponding standard curves below; two gels were analysed due to the number of conditions and replicates required. The number of worms analysed by 1D ^{1}H NMR spectroscopy was extrapolated from interpolated numbers.

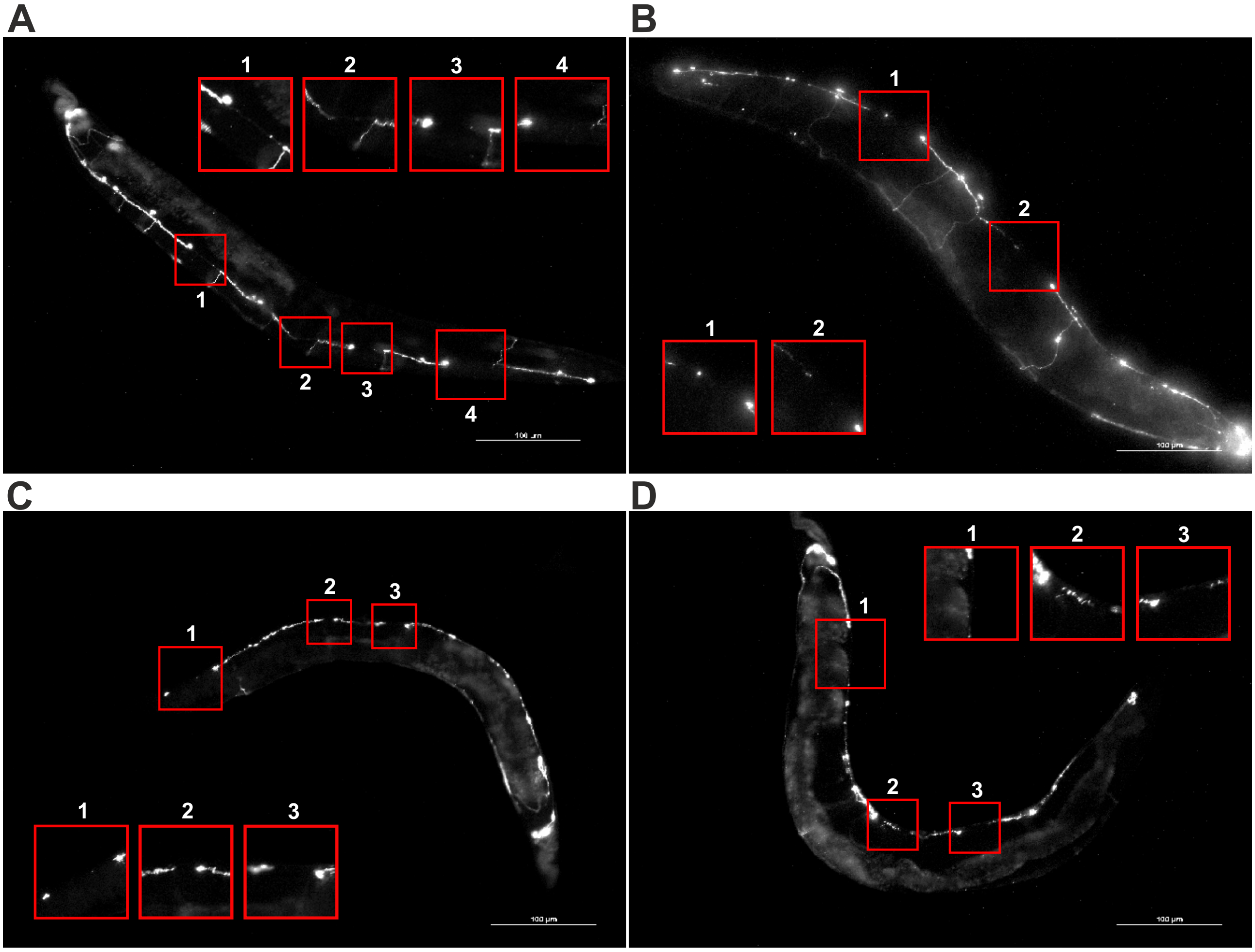

Figure S9. Representative images of day 3 *daf-16* null mutant TDP-43(A315T) worms expressing GFP in GABAergic neurons. Age-synchronised worms were chronically treated with 8mM (A) succinimide or (B) ethosuximide, (C) 0.4 % DMSO vehicle or (D) 0.05 mM MPS in 0.4 % DMSO from the L1 larval stage. Breaks within the D-type GABAergic motor neurons in the ventral nerve cord are indicated and enlarged with insets (red boxes). Images were taken at 200X magnification, scale bars = 100 µm.

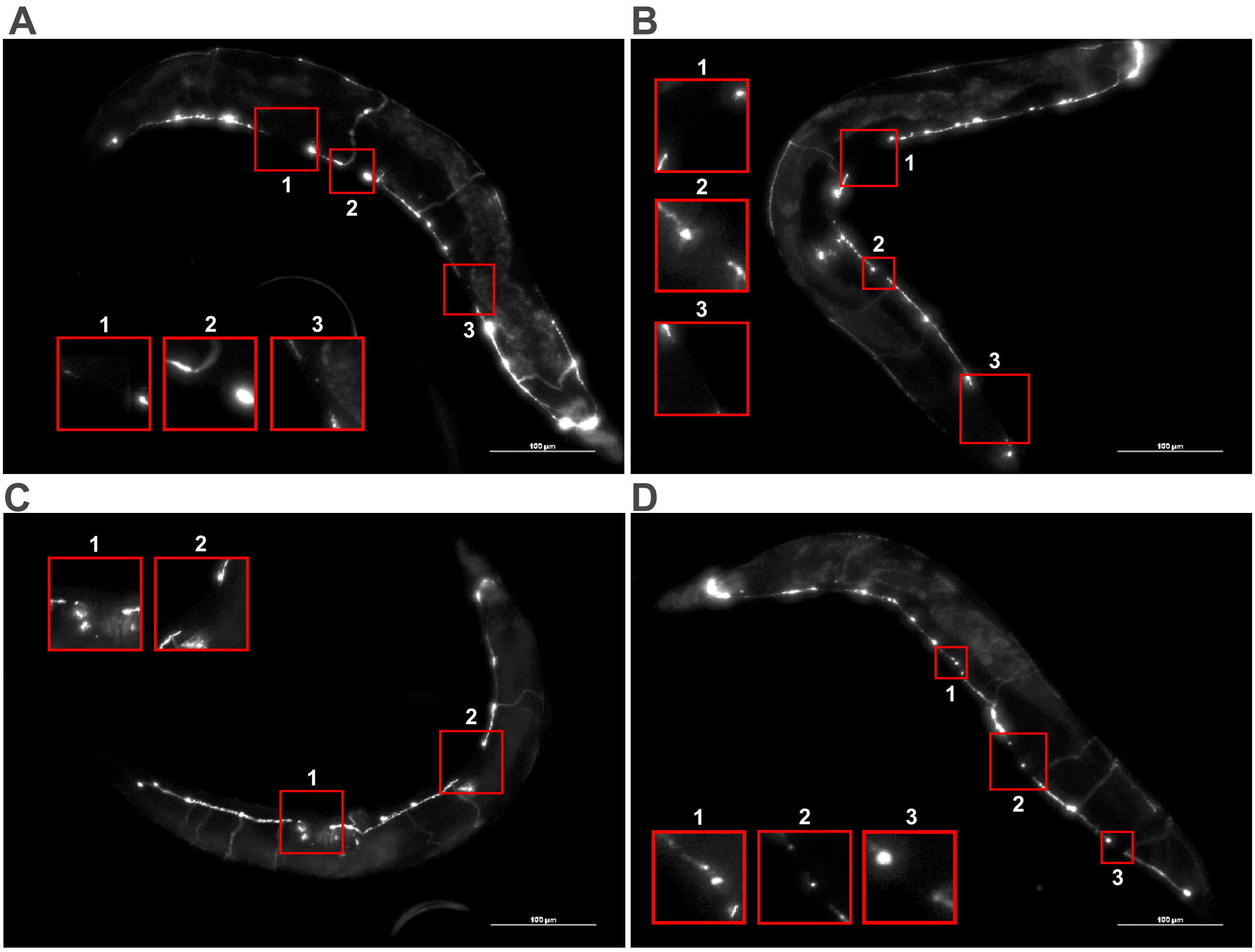

Figure S10. Representative images of day 5 *daf-16* null mutant TDP-43(A315T) worms expressing GFP in GABAergic neurons. Age-synchronised worms were chronically treated with 8mM (A) succinimide or (B) ethosuximide, (C) 0.4 % DMSO vehicle or (D) 0.05 mM MPS in 0.4 % DMSO from the L1 larval stage. Breaks within the D-type GABAergic motor neurons in the ventral nerve cord are indicated and enlarged with insets (red boxes). Images were taken at 200X magnification, scale bars = 100 µm.

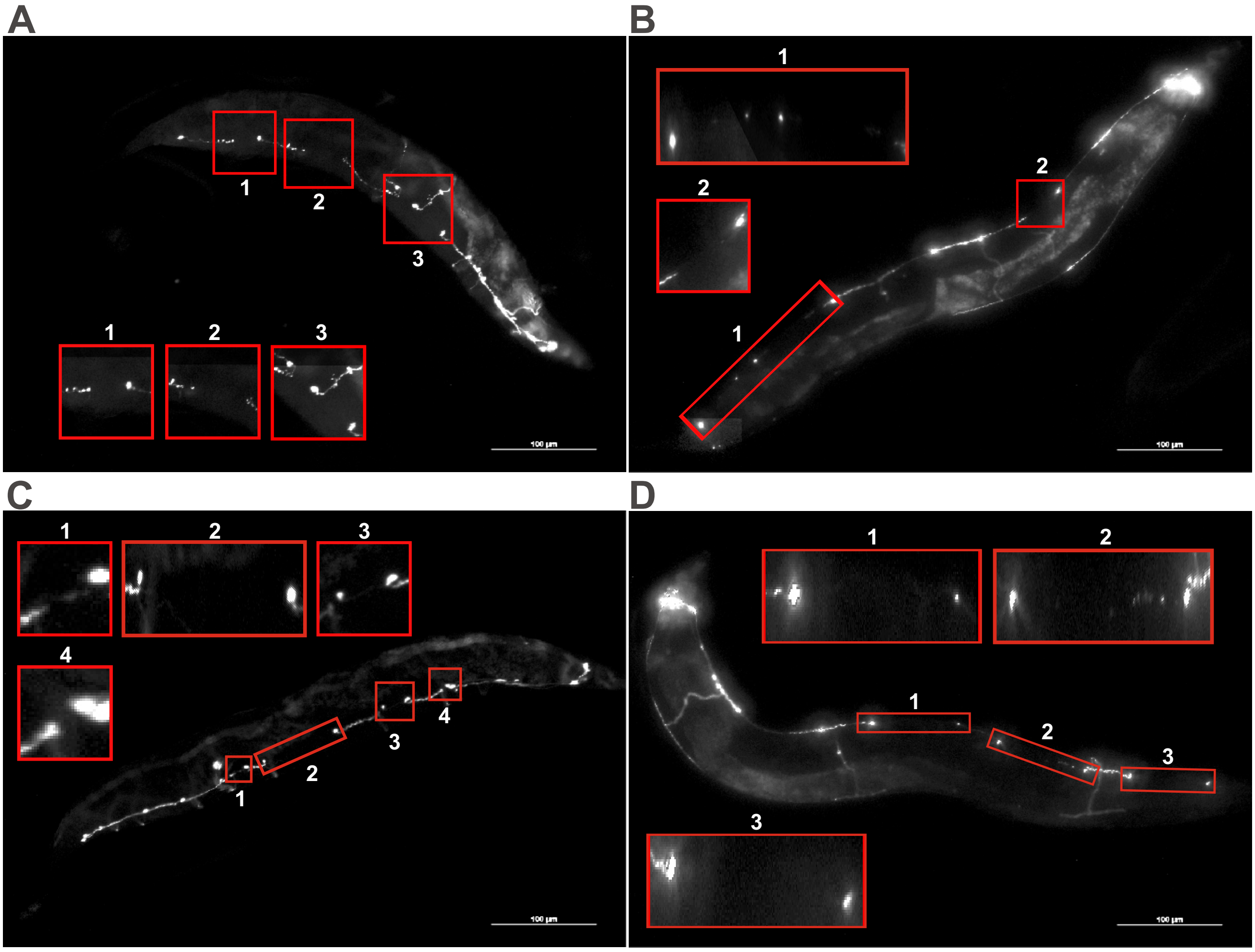

Figure S11. Representative images of day 7 *daf-16* null mutant TDP-43(A315T) worms expressing GFP in GABAergic neurons. Age-synchronised worms were chronically treated with 8mM (A) succinimide or (B) ethosuximide, (C) 0.4 % DMSO vehicle or (D) 0.05 mM MPS in 0.4 % DMSO from the L1 larval stage. Breaks within the D-type GABAergic motor neurons in the ventral nerve cord are indicated and enlarged with insets (red boxes). Images were taken at 200X magnification, scale bars = 100 µm.

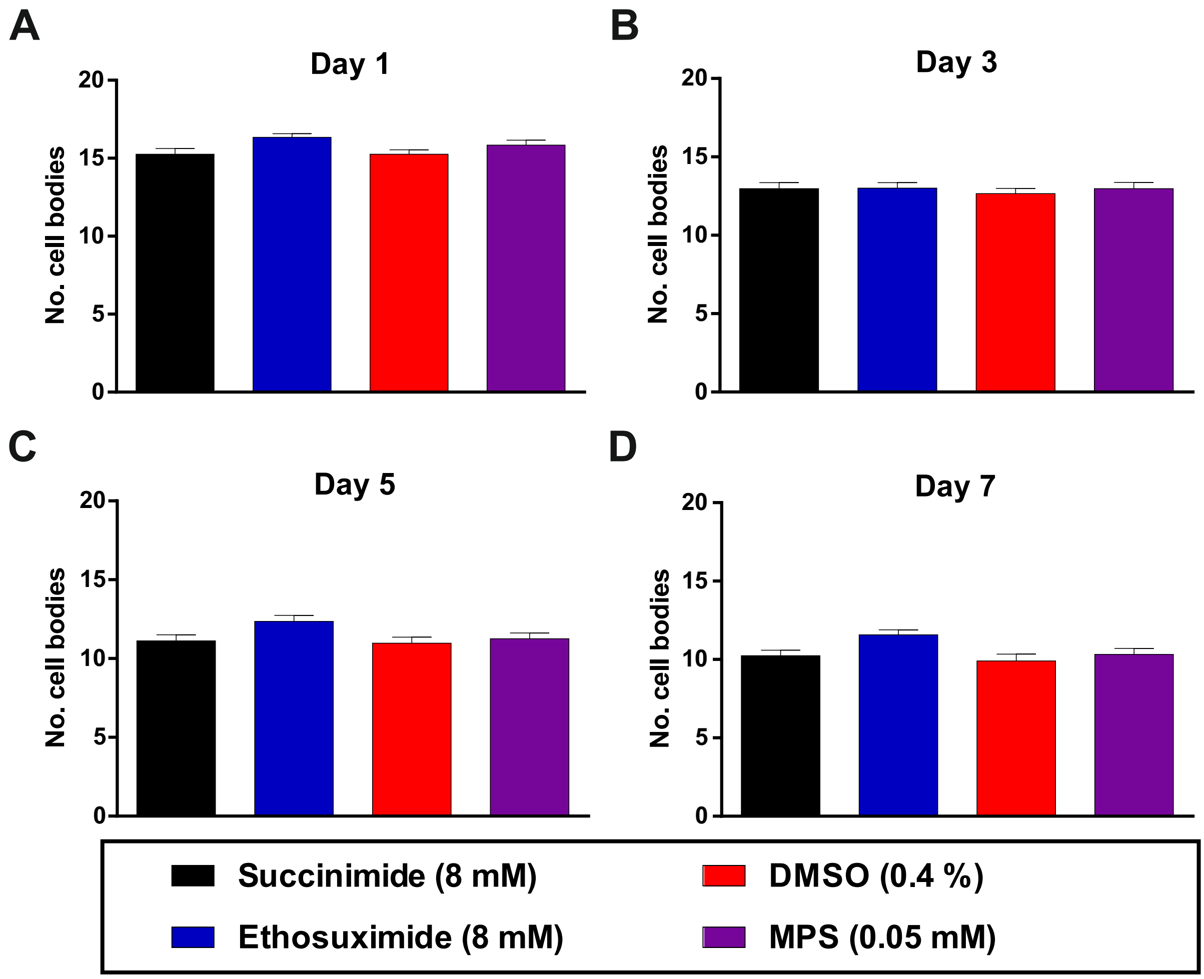

Figure S12. Compound 9-mediated protection against GABAergic neuronal cell body losses is DAF-16-dependent. The *daf-16* allele was deleted in the GABAergic GFP-expressing TDP-43(A315T) worms through crosses with a *daf-16* null mutant strain. Synchronised worms were chronically treated from the L1 larval stage and scored for the number of intact cell bodies from the D-type GABAergic motor neurons. The loss of DAF-16 completely abolished the ability of both ethosuximide and MPS to mitigate cell body losses at all ages. Data was pooled from four biological replicates, with comparisons performed via the Kruskal-Wallis test with Dunn's multiple comparisons against succinimide or DMSO controls ($n = 46 – 52$ worms per treatment per age point).

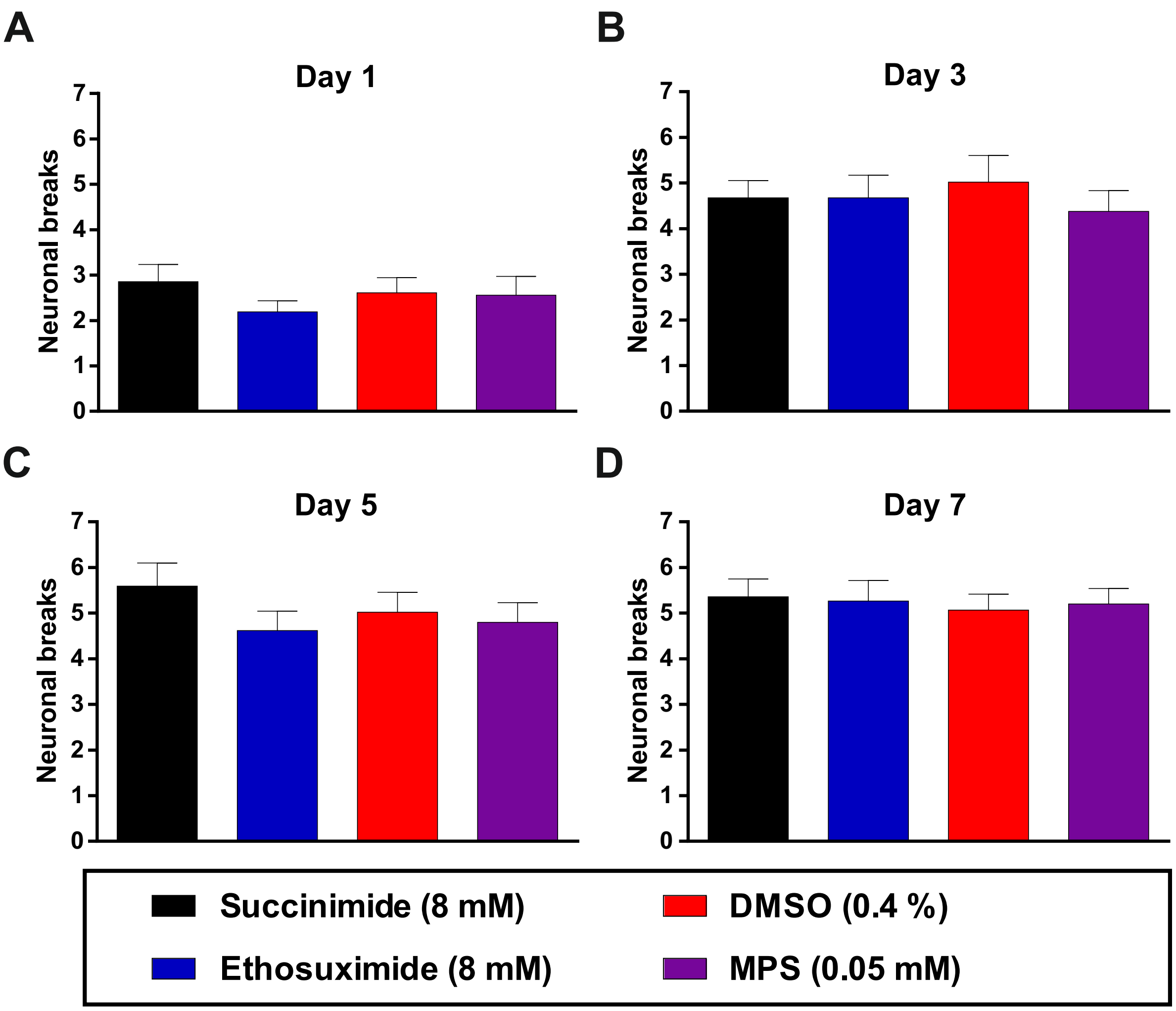

Figure S13. Protective effect of compound 9 against breaks in GABAergic neurons requires DAF-16. The *daf-16* allele was deleted in the GABAergic GFP-expressing TDP-43(A315T) worms through crosses with a *daf-16* null mutant strain. Synchronised worms were chronically treated from the L1 larval stage and assessed for breaks within the D-type GABAergic motor neurons. At all assayed ages, the loss of *daf-16* prevented reduction of neuronal breaks by both ethosuximide and MPS as compared to their respective succinimide and DMSO vehicle controls. Data was pooled from four biological replicates, with comparisons performed via the Kruskal-Wallis test with Dunn's multiple comparisons (n = 46 – 50 worms per treatment per age point).
